# *Taenia solium* taeniosis/cysticercosis and the co-distribution with schistosomiasis in Africa

**DOI:** 10.1186/s13071-015-0938-7

**Published:** 2015-06-12

**Authors:** Uffe Christian Braae, Christopher F. L. Saarnak, Samson Mukaratirwa, Brecht Devleesschauwer, Pascal Magnussen, Maria Vang Johansen

**Affiliations:** Department of Veterinary Disease Biology, Section for Parasitology and Aquatic Diseases, Faculty of Health and Medical Sciences, University of Copenhagen, DK-1870 Frederiksberg, Denmark; School of Life Sciences, University of KwaZulu-Natal, Durban, South Africa; Department of Virology, Parasitology and Immunology, Faculty of Veterinary Medicine, Ghent University, 9820 Merelbeke, Belgium; Institute of Health and Society (IRSS), Université catholique de Louvain, 1200 Brussels, Belgium; Centre for Medical Parasitology, Faculty of Health and Medical Sciences, University of Copenhagen, DK-1353 Copenhagen, Denmark

**Keywords:** African pig population, Co-distribution, Cysticercosis, Mapping, Prevalence, Schistosomiasis, *Taenia solium*

## Abstract

**Background:**

This study aimed to map the distribution of *Taenia solium* taeniosis/cysticercosis and the co-distribution with schistosomiasis in Africa. These two major neglected tropical diseases are presumed to be widely distributed in Africa, but currently the level of co-distribution is unclear.

**Methods:**

A literature search on *T. solium* taeniosis/cysticercosis was performed to compile all known studies on the presence of *T. solium* and apparent prevalence of taeniosis and porcine cysticercosis in Africa. Studies were geo-referenced using an online gazetteer. A Bayesian framework was used to combine the epidemiological data on the apparent prevalence with external information on test characteristics to estimate informed district-level prevalence of taeniosis and porcine cysticercosis. Districts with *T. solium* taeniosis/cysticercosis presence were cross-referenced with the Global Neglected Tropical Diseases Database for schistosomiasis presence.

**Results:**

The search strategies identified 141 reports of *T. solium* in Africa from 1985 to 2014 from a total of 476 districts in 29 countries, 20 with porcine cysticercosis, 22 with human cysticercosis, and 16 with taeniosis, in addition to 2 countries identified from OIE reports. All 31 countries were considered, on national scale, to have co-distribution with schistosomiasis. Presence of both parasites was confirmed in 124 districts in 17 countries. The informed prevalence of taeniosis and porcine cysticercosis were estimated for 14 and 41 districts in 10 and 13 countries, respectively.

**Conclusions:**

With the paucity of data, *T. solium* infection is grossly under-reported and expected to be more widespread than this study suggests. In areas where co-distribution occurs there is a need for increased emphasis on evaluation of integrated intervention approaches for these two helminth infections and allocation of resources for evaluating the extent of adverse effects caused by mass drug administration.

**Electronic supplementary material:**

The online version of this article (doi:10.1186/s13071-015-0938-7) contains supplementary material, which is available to authorized users.

## Background

The major neglected tropical diseases, *Taenia solium* taeniosis/cysticercosis and schistosomiasis caused by *Schistosoma mansoni* or *S. haematobium* are presumed to be widely distributed in Africa. *Taenia solium* taeniosis/cysticercosis has been reported as an emerging disease in different regions of Africa [1, 2], but currently the exact distribution remains unclear. Reported prevalences of *T. solium* taeniosis and cysticercosis in African countries are not extensive and are further complicated by the lack of ‘gold standard’ tests for diagnosis. Diagnosis has so far been performed using several diagnostic tests with varying sensitivity and specificity [3–5]. Therefore, estimating informed prevalence is important to determine the actual disease burden. Informed prevalence is an estimation of the true prevalence based on the apparent prevalence while factoring in the imperfections in sensitivity and specificity of the diagnostic tests used. The distribution of schistosomiasis in Africa has been more extensively investigated than *T. solium* taeniosis/cysticercosis and this has allowed for country level prevalence and risk estimation of schistosomiasis for all African countries [6].

The World Health Organization (WHO) is aiming for elimination of schistosomiasis by 2020 and the road map for elimination of *T. solium* taeniosis/cysticercosis is under consideration by the WHO [7]. The WHO strategy for schistosomiasis elimination is primarily mass drug administration (MDA) of preventive chemotherapy as the main intervention tool. The WHO advocates that MDA against schistosomiasis will reduce morbidity and decrease transmission, which might also carry the added benefit of controlling other infections in co-endemic areas such as *T. solium* taeniosis/cysticercosis [7]. A way forward for control of *T. solium* is integration with schistosomiasis control programmes. However, the potential benefit of an integrated control effort against the two parasites has yet to be evaluated. The anthelminthic drug used against schistosomiasis is praziquantel (PZQ) because of its safety profile, easy administration, and mild side-effects [7]. The current recommended dose of PZQ for treatment of schistosomiasis is 40 mg/kg as a single dose [8]. PZQ has proved highly efficacious against taeniosis at a dose of 5–10 mg/kg [9], and the drug can therefore be used against both parasites. However, the dose recommended for schistosomiasis treatment may increase the risk of seizures in people who are suffering from human cysticercosis where the larvae are lodged in the central nervous system (neurocysticercosis, NCC). Even a single dose, lower than that recommended for schistosomiasis treatment, has been reported to induce seizures [10]. Flisser and colleagues [11] reported suspected cases of NCC based on clinical signs following treatment with 5 mg/kg PZQ, and in a follow-up of 2452 participants subjected to an MDA using PZQ at 5 mg/kg where of 1.3 % reported complaints of severe headache after treatment. Although MDA has been widely applied for control of schistosomiasis in Africa, the safety of PZQ in MDA in communities where schistosomiasis and NCC coexist is yet to be systematically assessed.

The distribution of *T. solium* taeniosis/cysticercosis in Africa is unclear and up-to-date prevalence maps do not exist. The distribution of schistosomiasis is also to some extent uncertain, but through the work of the Global Neglected Tropical Diseases Database (GNTD; http://www.gntd.org), a prevalence map based on more than 20,000 locations can be created. The database is continuously updated with the goal to use the information for public health campaigns against schistosomiasis. With the launch of online virtual globes such as Google Earth, online gazetteers have become a useful tool for geo-referencing of locations and also disease distribution. The GNTD has used online gazetteers in order to geographically locate the distribution of schistosomiasis [12], which in turn have been utilised for modelling past and future distribution maps of infection [13–15]. This paper aims to compile the available information on *T. solium* taeniosis/cysticercosis in Africa and use the information to estimate the informed prevalence of taeniosis and porcine cysticercosis on a district level, and determine districts were co-distribution of *T. solium* taeniosis/cysticercosis and schistosomiasis occurs.

## Methods

### Literature search

The following data were included in this study 1) peer-reviewed studies of *T. solium* taeniosis/cysticercosis in Africa, 2) “grey literature” on *T. solium* taeniosis/cysticercosis presence in Africa which consisted of informally published written materials such as reports and theses, 3) official reports of national pig populations in Africa available through national census data and FAOSTAT [16], the statistical database of the Food and Agriculture Organization of the United Nations, 4) porcine cysticercosis reports from the World Organisation for Animal Health (OIE), and 5) the schistosomiasis prevalence map currently used by the WHO for assessing MDA intervals [17].

We performed a literature search using PubMed (http://www.ncbi.nlm.nih.gov/pubmed/) with date restriction from 01-01-1985 to 05-01-2015 using the following search term: (solium OR Tapeworm OR Taeniasis OR Taeni* OR Taeniosis OR Neurocysticercosis OR Cysticerc* OR cellulosae) AND (Algeria OR Angola OR Benin OR Botswana OR Burkina Faso OR Burundi OR Cameroon OR Central African Republic OR Chad OR Congo OR Zaire OR Cote d’Ivoire OR Ivory Coast OR Djibouti OR Egypt OR Equatorial Guinea OR Eritrea OR Ethiopia OR Gabon OR Gambia OR Ghana OR Guinea OR Guinea-Bissau OR Kenya OR Lesotho OR Liberia OR Libya OR Madagascar OR Malawi OR Mali OR Mauritania OR Morocco OR Mozambique OR Namibia OR Niger OR Nigeria OR Rwanda OR Senegal OR Sierra Leone OR Somalia OR South Africa OR South Sudan OR Sudan OR Swaziland OR Tanzania OR Togo OR Tunisia OR Uganda OR Zambia OR Zimbabwe). We also searched other databases such as Google Scholar (http://scholar.google.com), Thomson Reuter’s Web of Knowledge (http://www.wokinfo.com), Cab Direct (http://www.cabdirect.org), Société de Pathologie Exotique (http://www.pathexo.fr/), ProMED (http://www.isid.org), and African Journals Online (http://www.ajol.info) using the following keywords: “*Taenia solium*”, “porcine cysticercosis”, “*Cysticercus cellulosae*”, “neurocysticercosis”, “human cysticercosis”, “taeniosis”, and “taeniasis”. In addition, references found in suitable articles were also investigated to compile all known studies on presence of *T. solium* and prevalence of taeniosis and porcine cysticercosis.

### Data extraction

**Fig. 1:**
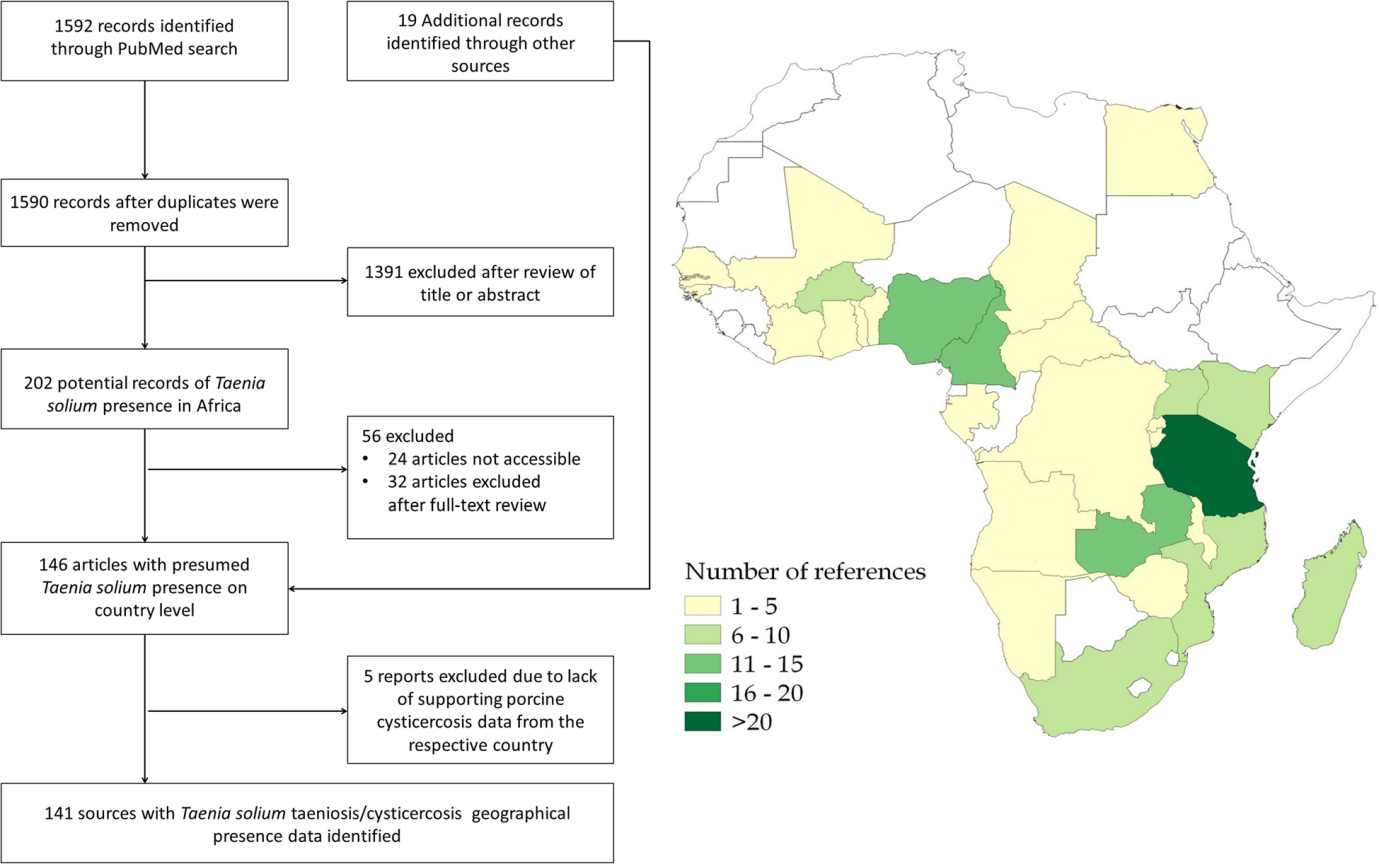
Diagram of literature search and countries where the studies were carried out

Presence of *T. solium* in this study was defined as a documented case of disease related to the *T. solium* tapeworm, whether it was diagnosed and documented as porcine cysticercosis, taeniosis, or human cysticercosis. Initially we reviewed all titles and abstracts, if accessible, and excluded studies from outside Africa, studies based on questionnaire only, environmental studies, and studies with no reference to geographical location. Authors of articles where full-text were inaccessible were contacted. The remaining studies were excluded if full-text was not available or if based on experimental studies where location of infection could not be established (Fig. 1). Studies on human cysticercosis were only included if the authors provided approximate location of where the patient presumably caught the infection. For example, Pönnighaus and colleagues [18] reported a case of cutaneous cysticercosis in Malawi, where it was beyond doubt that the disease had been acquired within the country. Other reports such as NCC cases suspected to be autochthonous but not confirmed were omitted. In order to reduce the risk of including *T. saginata* infections, studies reporting taeniosis, but without confirmation of the *T. solium* tapeworm, were only included if reports of porcine cysticercosis could be found for the respective country or if the OIE reported porcine cysticercosis to be present in the respective country.

*Taenia solium* taeniosis/cysticercosis cases were geo-referenced by extracting geographic information on the study quoted in the literature. If no geographic coordinates were provided by the authors, the geographic location was found by using the online gazetteer ‘Geonames’ (http://geonames.org). Distribution of schistosomiasis was extracted as point data from the GNTD and overlaid on the district-level occurrence of *T. solium* taeniosis/cysticercosis to determine districts with co-distribution.

**Fig. 2:**
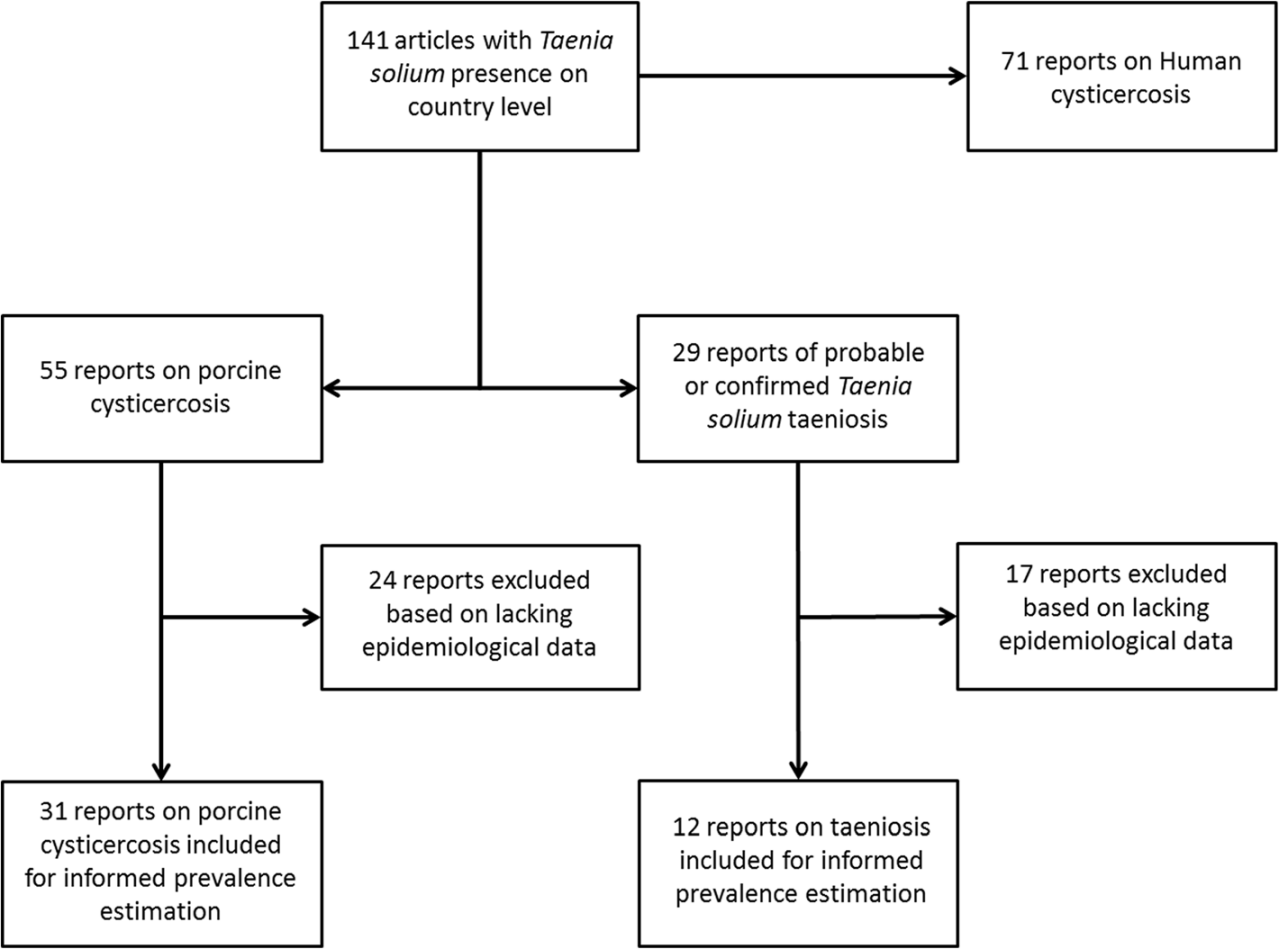
Flow chart of the selection of literature for the informed prevalence estimations

Informed district-level prevalence of taeniosis and porcine cysticercosis was estimated from apparent prevalence estimates extracted from the literature and external information on the sensitivity and specificity of the applied diagnostic tests. Data was only extracted if applied diagnostic tests, denominators and the number of positive subjects were provided. If multiple studies existed from the same second-level administrative division, the mapping was based on survey year (most recent), and then highest informed prevalence (Fig. 2). For studies were informed prevalence could be estimated based on multiple test assessment [19], this more informed estimate was preferred over the corresponding single test estimates. Studies with sample sizes of less than 30 individuals were excluded. Bayesian inference was used to obtain the informed prevalence estimates [20], using the functions in the R package prevalence version 0.3.0 [21]. The parameters for the probabilistic constraints in terms of sensitivity and specificity of the diagnostic tests used were obtained from key papers using the 95 % confidence intervals reported (Table 1). Further information and source code for both the single and multiple test informed prevalence assessments are available in the Additional file 1: Informed prevalence estimation.

**Table 1:** Parameters used for the probabilistic constraints for sensitivity and specificity of the different diagnostic tests using the 95 % confidence intervals reported in key papers

Test	Disease	Sensitivity (%)	Specificity (%)	Reference
Lingual examination	Porcine cysticercosis	16.1–21.0	90.0–100	[4]
Post-mortem	Porcine cysticercosis	22.1–38.7	90.0–100	[4]
Ag-ELISA (B158/B60)	Porcine cysticercosis	64.5–86.7	91.2–94.7	[4]
Ag-ELISA (HP10)	Porcine cysticercosis	52.7–84.7	44.6–85.1	[54, 55]
Coprology	Taeniosis	11.1–96.5	99.5–100^a^	[56]
Copro-Ag-ELISA	Taeniosis	61.9–98.0	90.0–93.8	[56]

^a^Test only genus specific

Data on the African pig population were extracted from national livestock census reports or FAOSTAT database if national census data were missing, and divided with the countries human population obtained from the UN to yield a per capita pig population [22]. Data on recent (2005 to 2014) reports of porcine cysticercosis that have been submitted to the OIE were extracted from the OIE database WAHID Interface [23]. Reports from the database are in 6 months intervals with disease status divided into 5 categories: disease was present, suspected but not confirmed, not reported during this period, never reported, and no information available. We have pooled the data into one ‘disease status’ during the period 2005 to 2014, ranking the five categories in the following order 1) disease was present, 2) suspected but not confirmed, 3) not reported during this period, 4) never reported, and 5) no information available.

## Results

The search strategies identified 141 reports of *T. solium* taeniosis/cysticercosis in Africa from 1985 to 2014, written in English, French, Portuguese, Italian, Danish, and German. The reports confirmed presence of *T. solium* taeniosis/cysticercosis in 476 second-level administrative units (i.e., districts) or equivalent from 29 African countries, with porcine cysticercosis reported in 20 countries, human cysticercosis reported in 22 countries, and taeniosis reported in 16 countries of which only 3 countries had studies confirming *T. solium* taeniosis cases (Table 2). No attempts were made to differentiate between *T. solium* and *T. saginata* infections in the reports from the remaining 13 countries. For additional 2 countries (Côte d’Ivoire [24] and Namibia [25]) totalling 31 countries, data were included based on OIE reports of porcine cysticercosis. This was for Côte d’Ivoire further supported by older literature documenting *T. solium* presence [26], but since neither studies on porcine cysticercosis nor human cysticercosis could be found in the literature search for Namibia, the presence of *T. solium* based on taeniosis is therefore uncertain. In Lesotho and Swaziland, no official information was found but *T. solium* presence in Lesotho has been mentioned in literature [27]. However, this was not deemed sufficient to be included in this study. There is a paucity of data from North Africa, but presumably due to the cultural/religious beliefs prevailing in the region, low prevalence could be expected. However, since pigs are kept in the region, we cannot assume the region is disease free.

**Table 2:** Presence of *Taenia solium* cysticercosis recorded in African countries from 1985 to 2014. *Taenia solium* taeniosis was not confirmed unless otherwise stated

Country	Porcine cysticercosis	Taeniosis	Human cysticercosis	No of References
Angola		[57]		1
Benin	[58]		[59–61]	4
Burkina Faso	[62]		[63–69]	8
Burundi	[70]	[70]	[41, 70–73]	5
Cameroon	[74–78]	[79, 80]^a^	[79, 81–87]	7
Central African Republic			[88]	1
Chad	[78]			1
Côte d’Ivoire		[24]		1
Democratic Republic of Congo	[89, 90]	[38]	[38]	3
Egypt	[91]			1
Gabon			[92]	1
Gambia	[93]			1
Ghana	[94]		[95]	2
Guinea-Bissau		[96, 97]		2
Kenya	[30, 98–100]		[95, 101]	6
Madagascar	[102–104]	[105]	[106–111]	10
Malawi			[18, 112]	2
Mali			[113]	1
Mozambique	[51, 114, 115]	[116]^a^	[116–118]	6
Namibia		[25]		1
Nigeria	[119–123]	[120, 123–127]	[40, 128, 129]	12
Rwanda			[130]	1
Senegal	[93]	[131, 132]	[132]	3
South Africa	[55]	[133, 134]	[95, 133, 135–140]	10
Tanzania	[28, 29, 31, 141–150]	[39, 151]^a^	[39, 95, 152–156]	21
Togo	[157]	[157]	[158–161]	5
Uganda	[162–167]	[168]	[95, 169]	9
Zambia	[4, 52, 170–172]	[36, 173–175]	[36, 42, 173, 174, 176, 177]	12
Zimbabwe	[178]		[179, 180]	3

^a^ Confirmed *Taenia solium* taeniosis cases

According to the ranking of OIE’s reports of porcine cysticercosis for 2005–2014, the disease was present in 18 African countries and additionally suspected in three: Equatorial Guinea, Kenya, and Tanzania. For both Kenya and Tanzania, literature confirms the presence of *T. solium* during this period [28–31], but no data exist from Equatorial Guinea. This indicates insufficient national reporting to the OIE. In 2 of the 18 countries with porcine cysticercosis according to the OIE, i.e., Congo and Niger, no other documentation of *T. solium* presence could be found.

**Fig. 3:**
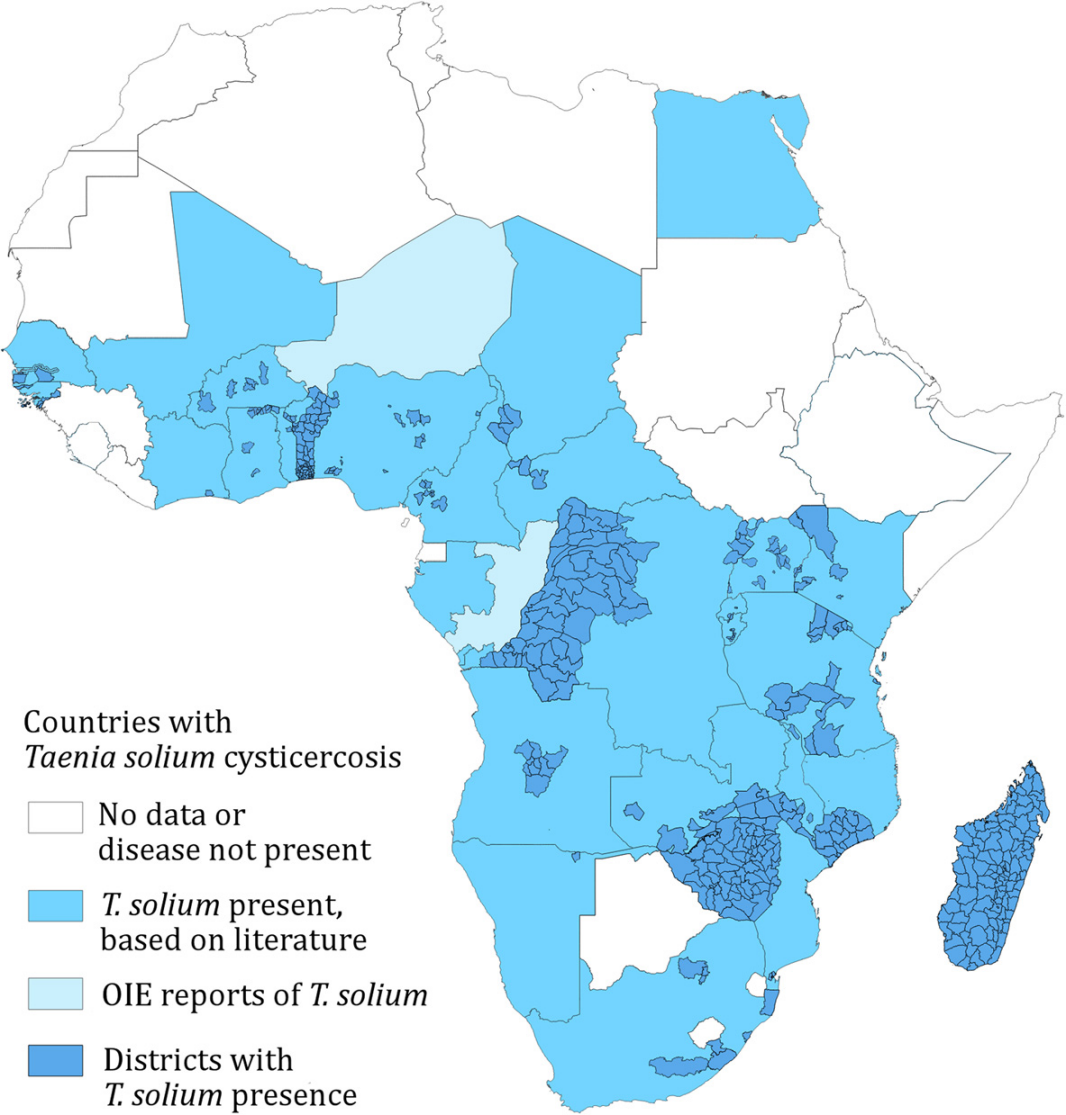
African countries and districts where *Taenia solium* taeniosis/cysticercosis has been confirmed from 1985 to 2014

**Fig. 4:**
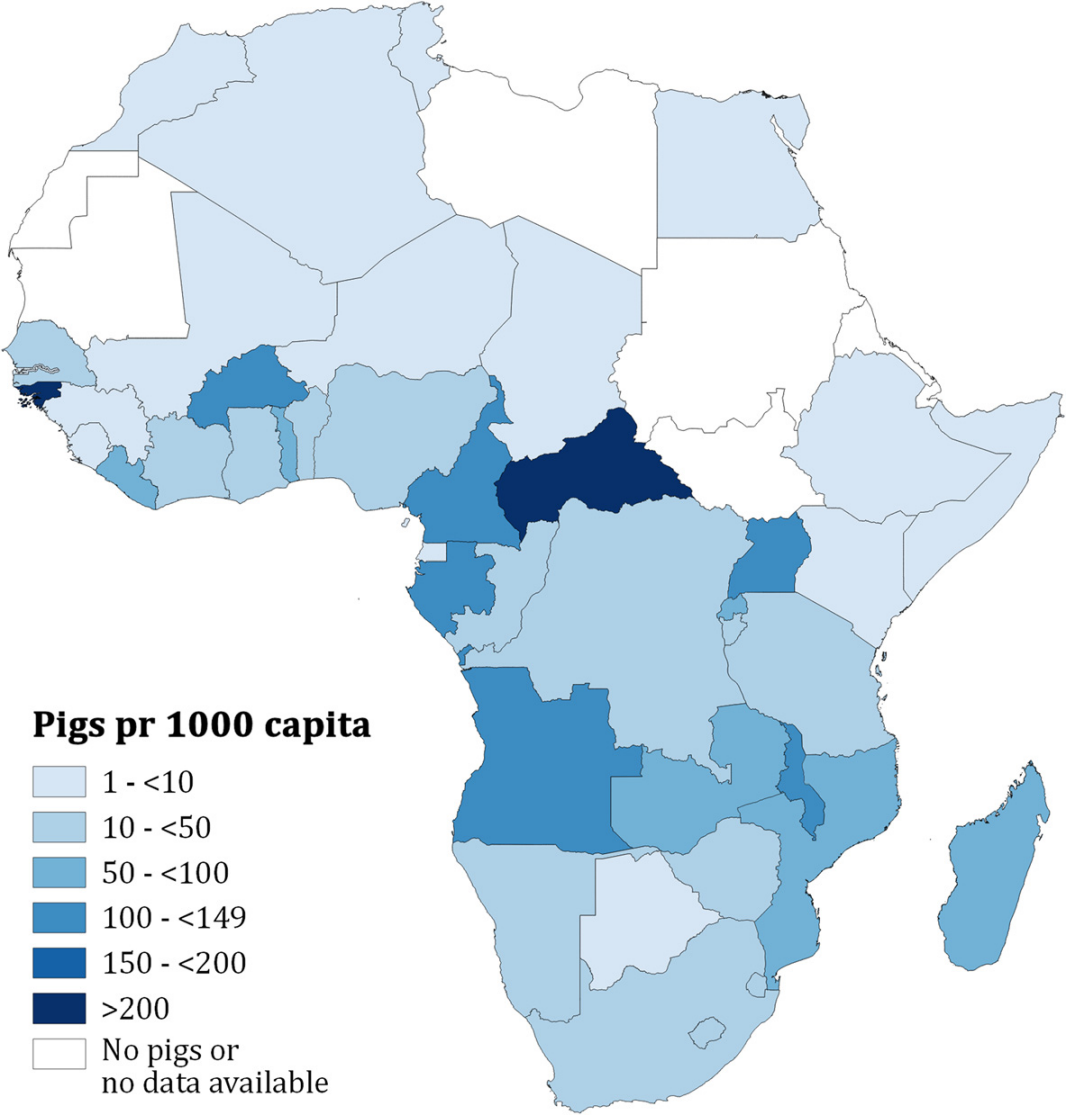
African pig population per capita on a national level in 2011. National pig populations were obtained from census reports or FAOSTAT

Figure 3 shows the presence of *T. solium* taeniosis/cysticercosis on national and district levels on the African continent and Madagascar. Data are not readily available for many African countries and in countries where data exist there are large areas in which prevalence is unknown. Importantly there are 10 African countries where pig keeping is known to take place, but in which no data exists for the presence of *T. solium*. Although, in three of these countries the pig population per capita is relatively low (Fig. 4).

**Fig. 5:**
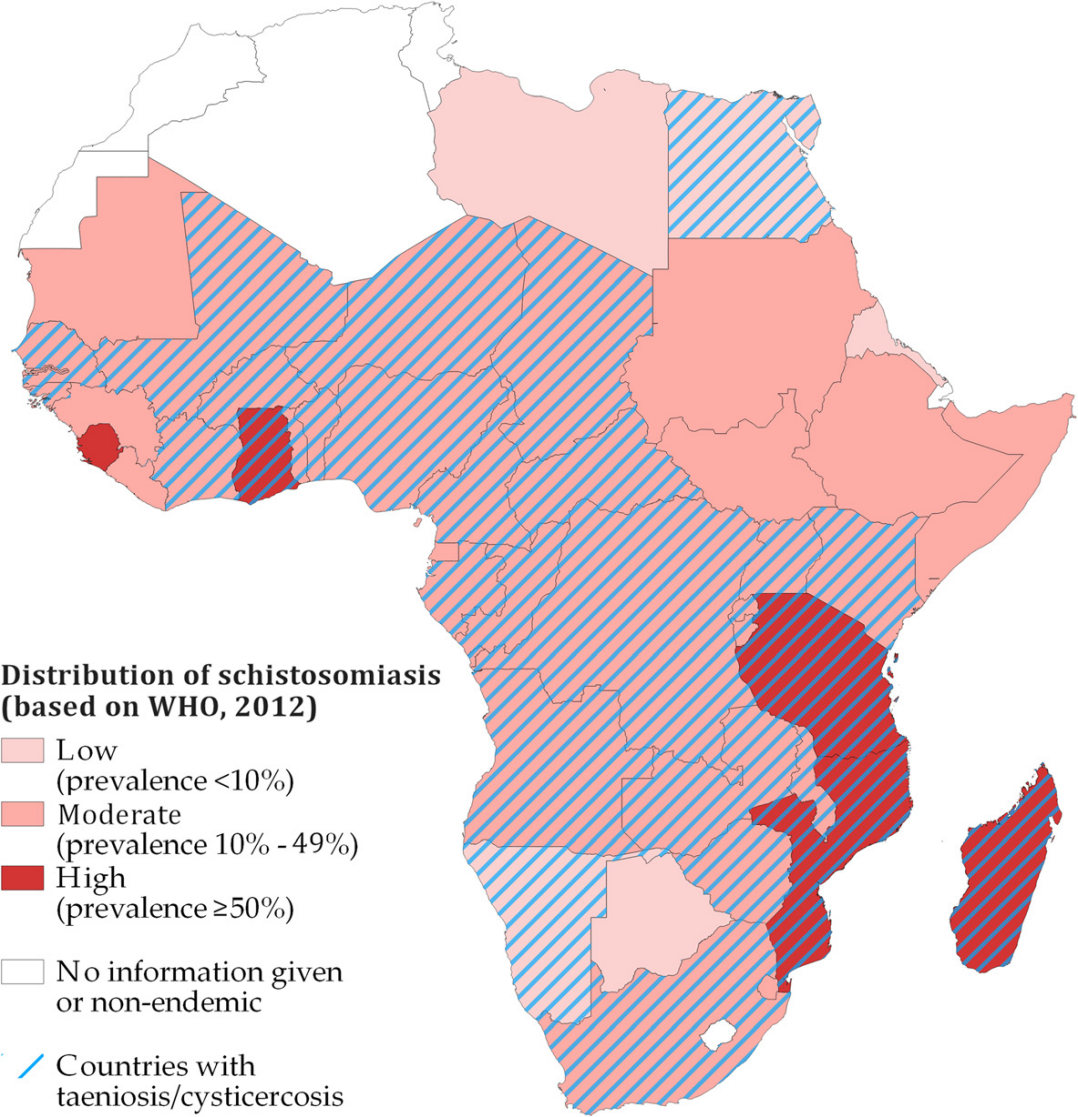
Co-distribution of *Taenia solium* infections in humans and/or pigs and schistosomiasis in Africa based on studies from 1985 to 2014

Figure 5 shows the presence of *T. solium* taeniosis/cysticercosis in Africa super-imposed onto African countries where the WHO recommended MDA against schistosomiasis with three different treatment intervals based on prevalence; high (≥50 %), medium (≥10 to <50 %), and low (<10 %). High prevalence areas fall under the recommendation of annual MDA of PZQ to all school-aged children and populations at risk. In medium prevalence areas biennially MDA of PZQ to all school-aged children are recommended, and in low prevalence areas all school-aged children should be treated twice during their schooling. In all 31 countries where *T. solium* taeniosis/cysticercosis was found, MDA was also recommended for control of schistosomiasis. *Taenia solium* has been confirmed in four countries (Ghana, Madagascar, Mozambique, and Tanzania) out of the five schistosomiasis high prevalence countries. No information is available for the fifth country (Sierra Leone) in terms of *T. solium* distribution, but pigs are present (Fig. 4). The WHO MDA recommendations represent an estimate of the mean schistosomiasis burden within a country. By overlaying the GNTD data on schistosomiasis with the district data on *T. solium* distribution, we found co-distribution in 124 out of the 476 districts were *T. solium* occurred. In these districts the GNTD data showed a mean schistosomiasis prevalence of 23.1 %, with a mean maximum prevalence of 49.6 %.

**Fig. 6:**
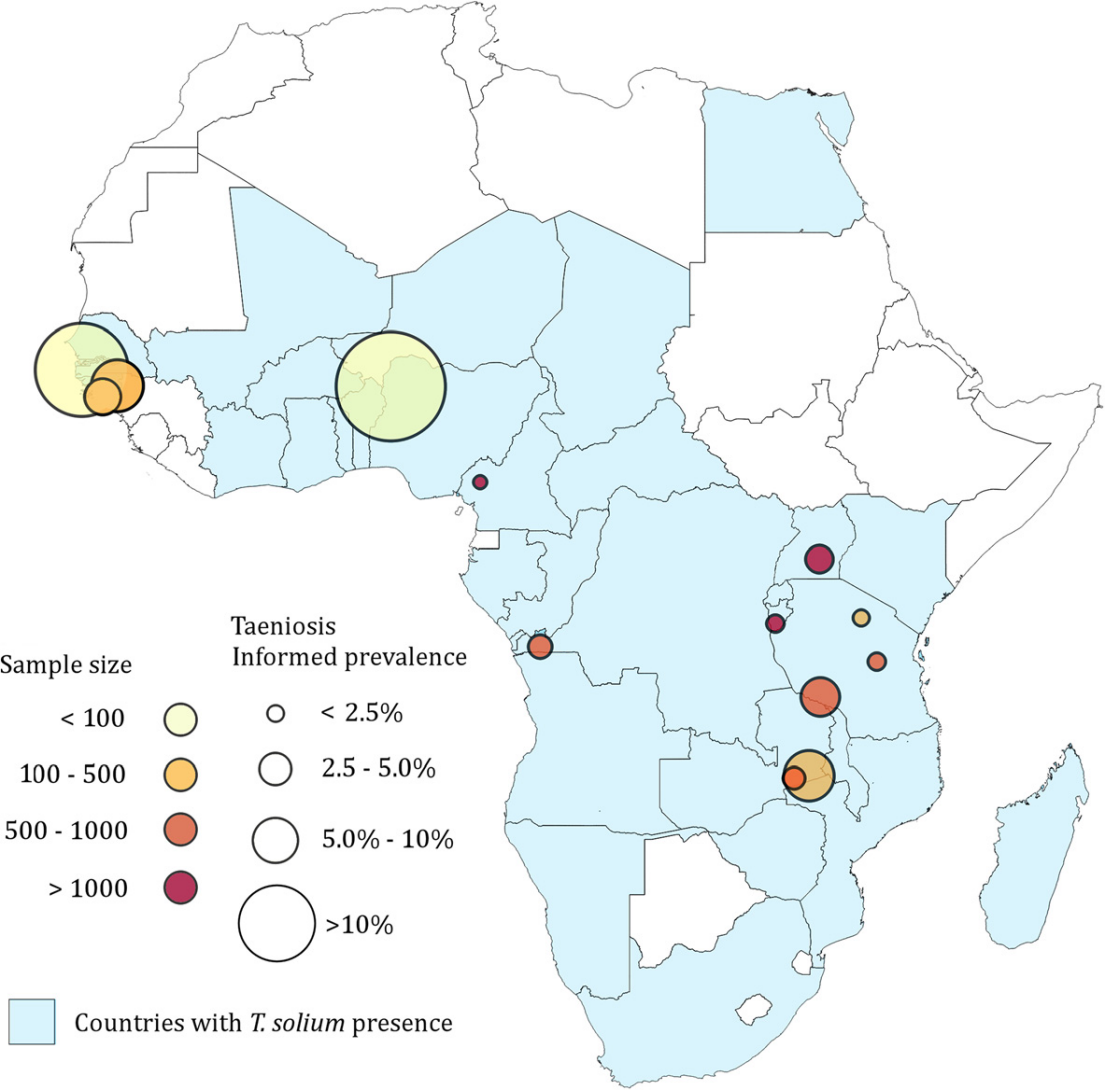
Informed prevalence of taeniosis in Africa from 1983 to 2010

**Fig. 7:**
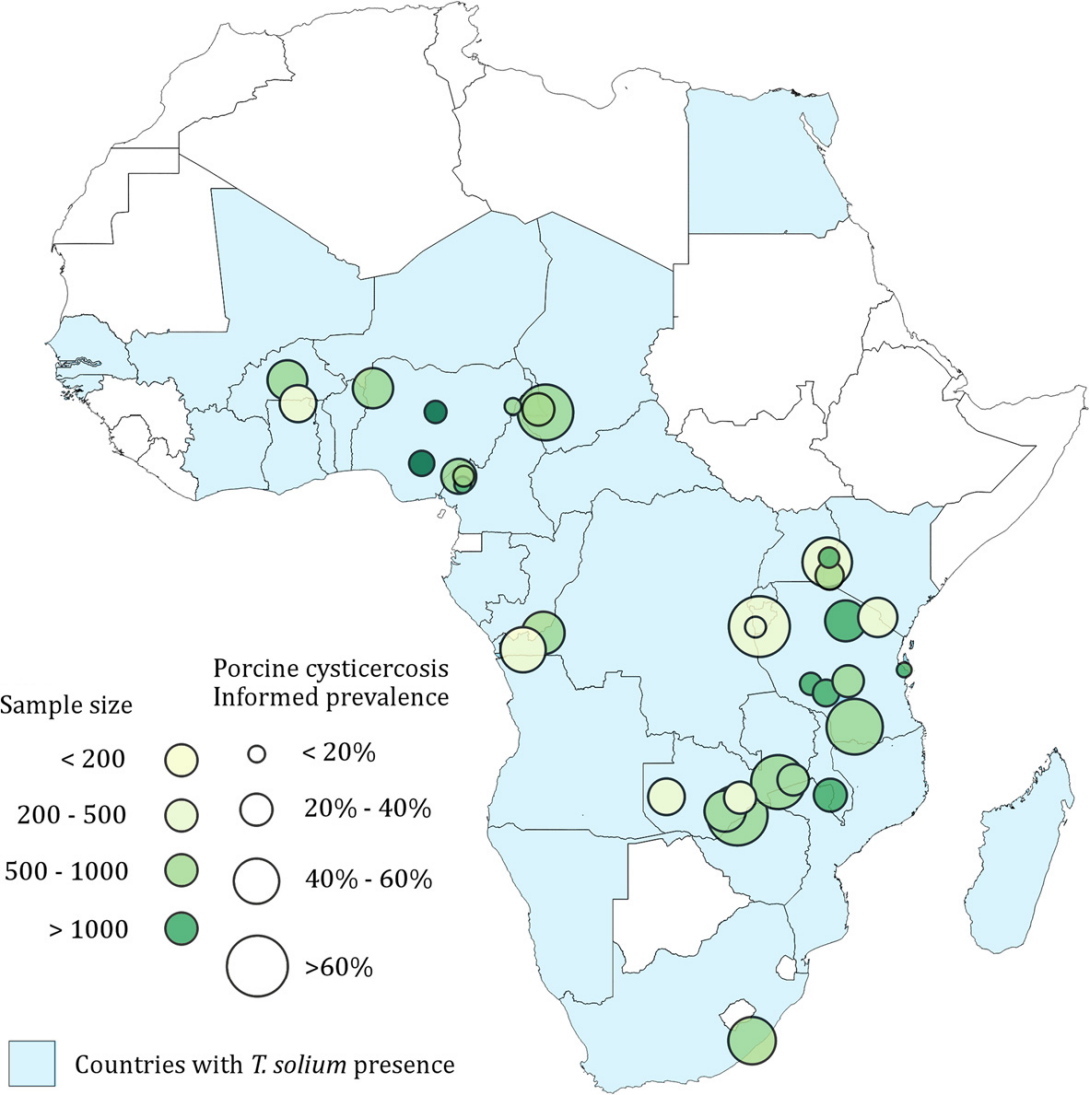
Informed prevalence of porcine cysticercosis in Africa from 1985 to 2013

Figures 6 and 7 represent the informed prevalence estimated for *T. solium* taeniosis and porcine cysticercosis, respectively, based on the studies that fulfilled the criteria for Bayesian inference (Table 3), and the selection criteria. Informed prevalence for taeniosis was calculated for 14 districts in 10 countries out of the 16 countries in which taeniosis were found (Fig. 6). For Côte d’Ivoire, Madagascar, Mozambique, Namibia, Togo, and Zimbabwe the literature did not contain the necessary epidemiological information and was excluded from the analysis. Informed prevalence for porcine cysticercosis was estimated for 41 districts in 13 out of the 20 countries in which porcine cysticercosis were confirmed (Fig. 7). Finally, detailed epidemiological data on porcine cysticercosis infection were missing from 7 (65 %) out of the 20 endemic countries based on the literature.

**Table 3:** Studies included in the calculation of informed prevalence of *Taenia solium* taeniosis and porcine cysticercosis by Bayesian inference

Country	Porcine cysticercosis	Taeniosis
Burkina Faso	[62]	
Burundi	[70]	[70]
Cameroon	[74–78]	[80]^a^
Chad	[78]	
Democratic Republic of Congo	[90]	[38]
Ghana	[94]	
Guinea-Bissau		[96, 97]
Kenya	[30, 98–100]	
Mozambique	[51]	
Nigeria	[119–121, 123]	[120]
Senegal		[132]
South Africa	[54]	[133]
Tanzania	[29, 31, 141, 142, 144, 149, 150]	[39, 151]^a^
Uganda	[167]	[168]
Zambia	[4, 170–172]	[36]

^a^ Cases of *Taenia solium* taeniosis confirmed

## Discussion

*Taenia solium* taeniosis/cysticercosis was confirmed in 476 districts in 29 African countries based on the literature search. According to the OIE reports from 2005 to 2014, the parasite is present in additional two countries, totalling 31 endemic countries in Africa. The findings correspond well to the WHO risk map for cysticercosis previously published [32], but have a much higher level of accuracy with distribution on district level in certain areas. With only 141 references identified from surveys in Africa, occurrence is probably grossly underreported. Several countries e.g. Guinea, Sierra Leone, and Liberia, with relatively large porcine populations still need to be investigated for *T. solium* presence, which emphasises the need for more research on disease distribution. However, this is complicated by the fact that *T. solium* has a focal distribution [33]. Pig keeping is far from evenly distributed across the African continent, nor within single countries, region or even districts.

In all 31 countries where *T. solium* occurrence was documented, schistosomiasis and *T. solium* taeniosis/cysticercosis can be considered to co-exist. Data on the distribution of *T. solium* is sparse on the district level and more data is essential to construct more accurate co-distribution maps, but nonetheless co-distribution was confirmed in 124 districts in 17 countries. Identifying co-endemic clusters on the same administrative level as MDA is carried out in the respective countries will enable identification of communities at risk of adverse effects from treatment with PZQ due to NCC. Even at village level significant variation in disease distribution is expected for both schistosomiasis and *T. solium* taeniosis/cysticercosis, as transmission of the disease is dependent on the presences of the respective intermediate hosts. This can result in large differences in disease prevalence within small geographical areas. However, since inadequate sanitation is an important risk factor for both parasites, frequent overlaps would be expected.

According to the WHO, preventive chemotherapy against schistosomiasis is required in some districts of all sub-Saharan countries, except Lesotho where the disease is not endemic [17]. More than 240 million people in Africa were in need of preventive chemotherapy against schistosomiasis in 2013 and just over 130 million of these were school-aged children [34]. School-aged children typically make-up a relatively large proportion of the total population within African countries and are considered the main group at risk for severe schistosomiasis morbidity and main contributors to egg excretion [35]. Taeniosis is however more equally distributed across age groups within the population [36], thus MDA of PZQ to school-aged children might therefore not be sufficient to significantly lower *Taenia* egg excretion within a community even though school-aged children make-up a large proportion of the population [37].

Currently there has been no monitoring or evaluation of MDA for schistosomiasis in communities where *T. solium* taeniosis/cysticercosis and schistosomiasis are co-distributed, which is highly warranted. Since the geographical distribution of *T. solium* remains to be fully elucidated in most African countries, the risk of adverse effects could be significantly underestimated. Several studies in Africa have confirmed the presence of human cysticercosis and specifically NCC in areas where schistosomiasis is endemic [36, 38–41]. Although NCC is considered more common in adults [42], children are also infected and in South Africa children down to the age of three have been found to suffer from NCC [43].

On-going control programmes in communities where pigs are kept or consumed, should be systematically monitored for adverse events that may incur from NCC in order to make precautionary implementations for future MDAs, such as determining prevalence of NCC in communities. Therefore, the number of pigs present in the communities, regions, and countries is an important key figure to obtain. Currently few African countries have performed a national census on livestock which makes the presumed rough estimates on national pig populations in Africa from FAOSTAT the only data available. However, the problem of NCC is not necessarily confined to areas where pigs are kept. Numerous accounts have shown that persons who neither raise pigs nor consume pork are also at risk of cysticercosis as people can accidently ingest *T. solium* eggs after coming into direct or indirect contact with tapeworm carriers, irrespective of their own cultural and religious practices or the presence of pigs [44–47]. Therefore, communication between the veterinary and public health sector is crucial for local authorities to get insight into the consequences of applying MDA to communities where schistosomiasis and *T. solium* taeniosis/cysticercosis are co-endemic.

Findings from the literature search indicate a discrepancy between the reports on the presence of porcine cysticercosis stated in the literature and that of which has been reported to the OIE. This may be due to porcine cysticercosis not being an international notifiable disease, which may lead to inconsistencies in reporting to the OIE by member states. There is a need to improve communication between the scientific community and OIE in addition to the respective member states from Africa to alleviate this problem.

The informed prevalences of taeniosis and porcine cysticercosis should be regarded as best estimates rather than absolute truths. Sensitivity and specificity are not necessarily intrinsic to the specific diagnostic test used, but affected by external factors [48], or by the intensity of the infection, which commonly vary among studies [49]. The Bayesian inference does however give the best possible comparison and thereby provides a rough overview of the disease burden within certain areas in the affected countries. Unfortunately, it does not provide any information on the *T. solium* cysticercosis burden for any entire nation. This requires more detailed nationwide epidemiological surveys [50].

Allowing pigs to roam freely is a well-known risk factor for porcine cysticercosis [51, 52], but production type and management were not considered in the study. Likewise, no differentiation was made between studies performed at slaughter slabs and farms. There is consensus that pigs in endemic African countries often are screened for cysticercosis by tongue examination before being sent for slaughter, resulting in higher apparent prevalence on farms compared to slaughter slabs. This causes bias in the surveys performed at slaughter slabs, which will underestimate the prevalence of porcine cysticercosis, because pigs with high intensity infections have been eliminated from the sample.

Pinpointing the origin of *T. solium* cases is difficult because taeniosis is often asymptomatic and symptoms of NCC often occur between 2 and 5 years after infection [53]. Thus, the subject would therefore require accurate long-term recollection of where and when the infection might have been contracted, and depending on travel history of the infected person the infection might not have been acquired at the same place as they were surveyed.

## Conclusion

Although *T. solium* is reported from the majority of African countries it is still grossly under-reported and for many areas the co-distribution with schistosomiasis on district level is still unknown. In areas where *T. solium* and schistosomiasis is co-distributed, an increased emphasis should be put on evaluating an integrated intervention approach for these two helminth infections. Resources should be allocated to evaluate the extent of adverse effects caused by the MDA of PZQ as preventive treatment in areas where people suffer from NCC. On-going control programmes should therefore be monitored, but reaching the goal of eliminating *T. solium* will require a One Health approach addressing both human and animal health.

## Additional file

Additional file 1:
**Informed prevalence estimation.** Estimating informed prevalence of *Taenia solium* taeniosis/cysticercosis in Africa.
