# Supplementary material for: Taenia solium taeniosis/cysticercosis and the co-distribution with schistosomiasis in Africa
Source: Parasit Vectors. 2015 Jun 12;8:323. doi: 10.1186/s13071-015-0938-7 (PMC4465723; doi:10.1186/s13071-015-0938-7)
Supplement: Additional file 1: — Informed prevalence estimation. Estimating informed prevalence of Taenia solium taeniosis/cysticercosis in Africa. [file 13071_2015_938_MOESM1_ESM.pdf]

Appendix

Estimating informed prevalence of *Taenia solium* taeniosis/cysticercosis in Africa

Uffe Christian Braae   Christopher F. L. Saarnak
Samson Mukaratirwa   Brecht Devleesschauwer   Pascal Magnussen
Maria Vang Johansen

February 25, 2015

Contents

1 Diagnostic test characteristics **2**

2 Apparent prevalence estimates **2**

3 Informed prevalence estimates **4**

3.1 Single test assessments 4
3.2 Multiple test assessments 7
3.2.1 Pouedet et al. 2002: Menoua division, Cameroon, 2000 7
3.2.2 Krecek et al. 2008, 2011: Eastern Cape province, South Africa, 2003 . . . 10
3.2.3 Dorny et al. 2004: Lusaka, Zambia, 2000 12

1 Diagnostic test characteristics

Sensitivity and specificity of the diagnostic tests were modelled as Uniform distributions. The lower and upper bounds for these distributions were obtained from key papers (Table 1). We excluded antibody assays as these tests measure exposure rather than active infection [1].

Table 1: Parameters used for the probabilistic constraints for sensitivity and specificity of the different diagnostic tests.

Test	Disease	Sensitivity (%)	Specificity (%)	Ref
Lingual examination	Porcine cysticercosis	16.1–21.0	90.0–100	[2]
Post-mortem	Porcine cysticercosis	22.1–38.7	90.0–100	[2]
Ag-ELISA (B158/B60)	Porcine cysticercosis	64.5–86.7	91.2–94.7	[2]
Ag-ELISA (HP10)	Porcine cysticercosis	52.7–84.7	44.6–85.1	[3, 4]
Coprology	Taeniosis	11.1–96.5	99.5–100	[5]
Copro-Ag-ELISA	Taeniosis	61.9–98.0	90.0–93.8	[5]

2 Apparent prevalence estimates

Apparent prevalence estimates for taeniosis and porcine cysticercosis are presented in Table 2 and Table 3. 95% exact binomial confidence intervals are calculated using the `propCI` function in the `prevalence` package version 0.3.0 [6].

Table 2: Apparent prevalence (AP) and corresponding 95% exact binomial confidence intervals (CI) for taeniosis in Africa

Ref	Country	Administrative division*	Year	Method	Examined	Positive	AP (%)	95% CI
[7]	Burundi	Rumonge	1994	Coprology	4717	12	0.3	0.1–0.4
[8]	Cameroon	Menoua	1999-2000	Coprology	3109	3	0.1	0.0–0.3
[9]	DR Congo	Cataractes	2009	Coprology	816	3	0.4	0.1–1.1
[10]	Guinea-Bissau	Cacine	1984	Coprology	203	1	0.5	0.0–2.7
[11]	Guinea-Bissau	Boé	1983	Coprology	289	5	1.7	0.6–4.0
[12]	Nigeria	Zuru	2008	Coprology	50	4	8.0	2.2–19.2
[13]	Senegal	Bignona	2009-2010	Coprology	43	2	4.7	0.6–15.8
[14]	South Africa	OR Tambo	1983-1984	Coprology	609	15	2.5	1.4–4.0
[15]	Tanzania	Kongwa	2008-2009	Coprology	929	1	0.1	0.0–0.6
[15]	Tanzania	Mbulu	2008-2009	Coprology	128	1	0.8	0.0–4.3
[16]	Tanzania	Mbozi	2009	Copro-Ag	820	43	5.2	3.8–7.0
[16]	Tanzania	Mbozi	2009	Coprology	820	9	1.1	0.5–2.1
[17]	Uganda	Kampala		Coprology	5313	36	0.7	0.5–0.9
[18]	Zambia	Petauke	2009	Copro-Ag	712	45	6.3	4.6–8.4
[18]	Zambia	Katete	2009-2010	Copro-Ag	226	27	11.9	8.0–16.9

**Administrative divisions are second-level administrative divisions*

Table 3: Apparent prevalence (AP) and corresponding 95% exact binomial confidence intervals (CI) for porcine cysticercosis in Africa

Ref	Country	Administrative division*	Year	Method	Examined	Positive	AP (%)	95% CI
[19]	Burkina Faso	Kadiogo	2007	Ag(B158/B60)	330	113	34	29–40
[7]	Burundi	Rumonge	1994	Post-mortem	50	1	2	0–11
[7]	Burundi	Buyengero	1994	Post-mortem	31	21	68	49–83
[20]	Cameroon	Mayo-Danay	1999	Lingual	441	68	15	12–19
[20]	Cameroon	Mayo-Danay	1999	Ag(B158/B60)	139	54	39	31–47
[21]	Cameroon	Mayo-Danay	2007-2008	Ag(B158/B60)	398	98	25	20–29
[22]	Cameroon	Mezam	2001	Lingual	499	18	4	2–6
[22]	Cameroon	Mezam	2001	Ag(B158/B60)	499	38	8	5–10
[23]	Cameroon	Menoua	2000	Lingual	707	43	6	4–8
[23]	Cameroon	Menoua	2000	Ag(B158/B60)	707	78	11	9–14
[24]	Cameroon	Momo	2001	Lingual	383	17	4	3–7
[24]	Cameroon	Momo	2001	Ag(B158/B60)	271	75	28	22–33
[20]	Chad	Mayo-Kebbi	1999	Ag(B158/B60)	125	51	41	32–50
[20]	Chad	Mayo-Kebbi	1999	Post-mortem	411	107	26	22–31
[25]	DR Congo	Kinshasa Urban	2009	Ag(B158/B60)	498	191	38	34–43
[25]	DR Congo	Cataractes	2009	Lingual	145	8	6	2–11
[25]	DR Congo	Cataractes	2009	Ag(B158/B60)	153	64	42	34–50
[26]	Ghana	Upper East§	1997	Post-mortem	60	7	12	5–23
[27]	Kenya	Homa Bay	2010	Lingual	392	22	6	4–8
[27]	Kenya	Homa Bay	2010	Ag(HP10)	232	76	33	27–39
[28]	Kenya	Busia	2005	Lingual	107	15	14	8–22
[29]	Kenya	Busia	2010	Ag(B158/B60)	284	11	4	2–7
[30]	Kenya	Teso	2003-2004	Lingual	505	33	7	5–9
[31]	Mozambique	Angónia	2007	Lingual	661	84	13	10–15
[31]	Mozambique	Angónia	2007	Ag(B158/B60)	661	231	35	31–39
[32]	Nigeria	Adamawa	2012	Post-mortem	247	8	3	1–6
[12]	Nigeria	Zuru	2008	Lingual	205	12	6	3–10
[12]	Nigeria	Zuru	2008	Post-mortem	118	17	14	9–22
[33]	Nigeria	Taraba	2010-2012	Post-mortem	4380	274	6	6–7
[34]	Nigeria	Nsukka	1985-1988	Lingual	1300	72	6	4–7
[3, 4]	South Africa	Eastern Cape§	2003	Lingual	261	31	12	8–16
[3, 4]	South Africa	Eastern Cape§	2003	Ag(HP10)	261	143	55	49–61
[3, 4]	South Africa	Eastern Cape§	2003	Ag(B158/B60)	261	89	34	28–40
[35]	Tanzania	Moshi Urban	1992	Post-mortem	60	8	13	6–25
[36]	Tanzania	Chunya	1999-2000	Lingual	722	55	8	6–10
[36]	Tanzania	Iringa Rural	1999-2000	Lingual	808	68	8	7–11
[36]	Tanzania	Songea & Mbinga	1999-2000	Lingual	302	51	17	13–22
[37]	Tanzania	Mbozi & Mbeya	2013	Ag(B158/B60)	998	202	20	18–23
[38]	Tanzania	Mbozi	2007-2008	Lingual	300	35	12	8–16
[38]	Tanzania	Mbozi	2007-2008	Ag(B158/B60)	300	96	32	27–38
[38]	Tanzania	Mbeya Rural	2007-2008	Lingual	300	18	6	4–9
[38]	Tanzania	Mbeya Rural	2007-2008	Ag(B158/B60)	300	92	31	25–36
[39]	Tanzania	Kinondoni, Ilala & Temeke	2007-2008	Post-mortem	731	43	6	4–8
[40]	Tanzania	Mbulu	1998	Lingual	770	134	17	15–20
[41]	Tanzania	Iringa Rural	2012	Lingual	308	23	7	5–11
[42]	Uganda	Kamuli & Kaliro	2002-2005	Ag(B158/B60)	480	41	9	6–11
[2]	Zambia	Lusaka	2000	Lingual	868	115	13	11–16

Table 3: (*Continued*)

Ref	Country	Administrative division*	Year	Method	Examined	Positive	AP (%)	95% CI
[2]	Zambia	Lusaka	2000	Post-mortem	868	121	14	12–16
[2]	Zambia	Lusaka	2000	Ag(B158/B60)	868	387	45	41–48
[43]	Zambia	Lusaka	2000	Lingual	1316	143	11	9–13
[43]	Zambia	Sinda	2001	Lingual	151	8	5	2–10
[43]	Zambia	Sinda	2001	Ag(B158/B60)	151	14	9	5–15
[43]	Zambia	Kalomo	2000	Lingual	98	8	8	4–15
[43]	Zambia	Kalomo	2000	Ag(B158/B60)	98	20	20	13–30
[44]	Zambia	Lusaka	2002-2003	Post-mortem	30	2	7	1–22
[44]	Zambia	Katete & Petauke	2002-2003	Post-mortem	35	10	29	15–46
[44]	Zambia	Katete & Petauke	2002-2003	Lingual	35	5	14	5–30
[45]	Zambia	Gwembe	2002-2003	Lingual	385	83	22	18–26
[45]	Zambia	Monze	2002-2003	Lingual	387	43	11	8–15
[45]	Zambia	Petauke	2002-2003	Lingual	384	25	7	4–9
[45]	Zambia	Katete	2002-2003	Lingual	385	29	8	5–11
[45]	Zambia	Mongu	2002-2003	Lingual	150	11	7	4–13
[45]	Zambia	Gwembe	2002-2003	Ag(B158/B60)	385	131	34	29–39
[45]	Zambia	Monze	2002-2003	Ag(B158/B60)	387	88	23	19–27
[45]	Zambia	Petauke	2002-2003	Ag(B158/B60)	384	56	15	11–19
[45]	Zambia	Katete	2002-2003	Ag(B158/B60)	385	74	19	15–24
[45]	Zambia	Mongu	2002-2003	Ag(B158/B60)	150	45	30	23–38

**Administrative divisions are second-level administrative divisions or first-level administrative divisions (indicated by §)*

3 Informed prevalence estimates

3.1 Single test assessments

The epidemiological data on apparent prevalence (Table 2, 3) were combined with external information on sensitivity and specificity of the applied diagnostics tests (Table 1) in a Bayesian framework to generate *informed* or *true* prevalence estimates [46]. Uncertainty in the test sensitivities and specificities were modelled as Uniform distributions with the ranges given in Table 1. A Beta(1,1) prior was assumed for the informed prevalence estimates. The general form of the applied Bayesian model is given by:

```
model {
  x ~ dbin(AP, n)
  AP <- SE * TP + (1 - SP) * (1 - TP)
  SE ~ dunif(se_min, se_max)
  SP ~ dunif(sp_min, sp_max)
  TP ~ dbeta(1, 1)
}
```

The informed prevalence estimates were obtained using the `truePrev` function in the `prevalence` package version 0.3.0 [6]. Per assessment, we generated two chains of 100,000 iterations, of which the first 50,000 were discarded as burn-in. Convergence was assessed by calculating the Brooks-Gelman-Rubin statistic.

Table 4 shows the informed prevalence estimates for taeniosis, and Table 5 shows the informed prevalence estimates for porcine cysticercosis.

Table 4: Informed prevalence (IP) estimates and corresponding 95% uncertainty intervals (UI) for taeniosis in Africa.

Country	Administrative division*	Year	Method	IP (%)	95% UI
Burundi	Rumonge	1994	Coprology	0.6	0.0–2.0
Cameroon	Menoua	1999-2000	Coprology	0.3	0.0–1.2
DR Congo	Cataractes	2009	Coprology	1.2	0.0–4.9
Guinea-Bissau	Cacine	1984	Coprology	3.1	0.1–12.8
Guinea-Bissau	Boé	1983	Coprology	6.8	1.0–21.4
Nigeria	Zuru	2008	Coprology	32.0	5.6–87.0
Senegal	Bignona	2009-2010	Coprology	23.0	2.5–75.5
South Africa	OR Tambo	1983-1984	Coprology	8.7	2.0–23.8
Tanzania	Kongwa	2008-2009	Coprology	0.6	0.0–2.7
Tanzania	Mbulu	2008-2009	Coprology	5.0	0.2–20.3
Tanzania	Mbozi	2009	Copro-Ag	0.6	0.0–2.0
Tanzania	Mbozi	2009	Coprology	3.6	0.5–11.0
Uganda	Kampala		Coprology	1.7	0.2–5.0
Zambia	Petauke	2009	Copro-Ag	1.0	0.0–3.1
Zambia	Katete	2009-2010	Copro-Ag	6.3	0.8–14.0

**Administrative divisions are second-level administrative divisions*

Table 5: Informed prevalence (IP) estimates and corresponding 95% uncertainty intervals (UI) for porcine cysticercosis in Africa.

Country	Administrative division*	Year	Method	IP (%)	95% UI
Burkina Faso	Kadiogo	2007	Ag(B158/B60)	40	31–52
Burundi	Rumonge	1994	Post-mortem	11	0–36
Burundi	Buyengero	1994	Post-mortem	94	77–100
Cameroon	Mayo-Danay	1999	Lingual	74	42–98
Cameroon	Mayo-Danay	1999	Ag(B158/B60)	47	34–63
Cameroon	Mayo-Danay	2007-2008	Ag(B158/B60)	26	19–35
Cameroon	Mezam	2001	Lingual	11	1–26
Cameroon	Mezam	2001	Ag(B158/B60)	2	0–6
Cameroon	Menoua	2000	Lingual	19	1–39
Cameroon	Menoua	2000	Ag(B158/B60)	6	2–11
Cameroon	Momo	2001	Lingual	14	1–33
Cameroon	Momo	2001	Ag(B158/B60)	31	22–41
Chad	Mayo-Kebbi	1999	Ag(B158/B60)	50	36–67
Chad	Mayo-Kebbi	1999	Post-mortem	78	55–99

Table 5: (*Continued*)

Country	Administrative division*	Year	Method	IP (%)	95% UI
DR Congo	Kinshasa Urban	2009	Ag(B158/B60)	46	37–58
DR Congo	Cataractes	2009	Lingual	21	1–51
DR Congo	Cataractes	2009	Ag(B158/B60)	52	38–68
Ghana	Upper East§	1997	Post-mortem	34	3–77
Kenya	Homa Bay	2010	Lingual	18	1–39
Kenya	Homa Bay	2010	Ag(HP10)	20	1–46
Kenya	Busia	2005	Lingual	62	14–97
Kenya	Busia	2010	Ag(B158/B60)	1	0–4
Kenya	Teso	2003-2004	Lingual	21	1–43
Mozambique	Angónia	2007	Lingual	55	21–84
Mozambique	Angónia	2007	Ag(B158/B60)	41	33–51
Nigeria	Adamawa	2012	Post-mortem	7	0–19
Nigeria	Zuru	2008	Lingual	21	1–48
Nigeria	Zuru	2008	Post-mortem	41	9–78
Nigeria	Taraba	2010-2012	Post-mortem	12	1–24
Nigeria	Nsukka	1985-1988	Lingual	16	1–34
South Africa	Eastern Cape§	2003	Lingual	50	10–87
South Africa	Eastern Cape§	2003	Ag(HP10)	54	5–97
South Africa	Eastern Cape§	2003	Ag(B158/B60)	40	30–52
Tanzania	Moshi Urban	1992	Post-mortem	39	5–84
Tanzania	Chunya	1999-2000	Lingual	24	1–48
Tanzania	Iringa Rural	1999-2000	Lingual	26	2–52
Tanzania	Songea & Mbinga	1999-2000	Lingual	80	47–99
Tanzania	Mbozi & Mbeya	2013	Ag(B158/B60)	20	14–26
Tanzania	Mbozi	2007-2008	Lingual	48	9–84
Tanzania	Mbozi	2007-2008	Ag(B158/B60)	37	28–48
Tanzania	Mbeya Rural	2007-2008	Lingual	20	1–44
Tanzania	Mbeya Rural	2007-2008	Ag(B158/B60)	35	26–46
Tanzania	Kinondoni, Ilala & Temeke	2007-2008	Post-mortem	11	1–25
Tanzania	Mbulu	1998	Lingual	85	61–99
Tanzania	Iringa Rural	2012	Lingual	25	2–53
Uganda	Kamuli & Kaliro	2002-2005	Ag(B158/B60)	3	0–8
Zambia	Lusaka	2000	Lingual	59	28–86
Zambia	Lusaka	2000	Post-mortem	37	15–60
Zambia	Lusaka	2000	Ag(B158/B60)	56	46–67
Zambia	Lusaka	2000	Lingual	39	7–66
Zambia	Sinda	2001	Lingual	20	1–49

Table 5: (*Continued*)

Country	Administrative division*	Year	Method	IP (%)	95% UI
Zambia	Sinda	2001	Ag(B158/B60)	5	0–13
Zambia	Kalomo	2000	Lingual	34	2–79
Zambia	Kalomo	2000	Ag(B158/B60)	21	9–34
Zambia	Lusaka	2002-2003	Post-mortem	26	1–74
Zambia	Katete & Petauke	2002-2003	Post-mortem	74	34–99
Zambia	Katete & Petauke	2002-2003	Lingual	59	8–98
Zambia	Gwembe	2002-2003	Lingual	92	75–100
Zambia	Monze	2002-2003	Lingual	43	7–77
Zambia	Petauke	2002-2003	Lingual	21	1–45
Zambia	Katete	2002-2003	Lingual	25	1–52
Zambia	Mongu	2002-2003	Lingual	28	2–64
Zambia	Gwembe	2002-2003	Ag(B158/B60)	40	31–51
Zambia	Monze	2002-2003	Ag(B158/B60)	23	16–32
Zambia	Petauke	2002-2003	Ag(B158/B60)	11	6–18
Zambia	Katete	2002-2003	Ag(B158/B60)	18	12–26
Zambia	Mongu	2002-2003	Ag(B158/B60)	34	23–48

**Administrative divisions are second-level administrative divisions or first-level administrative divisions (indicated by §)*

3.2 Multiple test assessments

In three studies, porcine samples were subjected to multiple tests and individual-level results were presented [2–4, 23]. Such datasets allowed estimating informed prevalence using the multi-test model introduced by Berkvens et al. [47]. This method is available in the `prevalence` package through the `truePrevMulti` function [6]. By default, 2 chains are generated of 20,000 posterior samples each, of which the first 10,000 are discarded as burn-in. In the following subsections, we repeat the analyses performed in the respective papers, but applying the test characteristics specified in Table 1 for consistency with the informed prevalence estimates presented above.

3.2.1 Pouedet et al. 2002: Menoua division, Cameroon, 2000

Pouedet et al. [23] examined 707 pigs in the Bafou and Bamendou communities of Menoua division, Cameroon, in 2000. They used three tests: lingual examination, Ag-ELISA (B158/B60) and Antibody ELISA. In this analysis, only the results of the first two tests are considered. Table 6 shows the obtained joint test results.

Table 6: Results of the study on porcine cysticercosis in Menoua division, Cameroon, 2000 ($n = 707$) [23]

Lingual examination	Ag-ELISA (B158/B60)	x
+	+	34
+	−	9
−	+	44
−	−	620

Table 7 shows the results of the informed prevalence assessments

Table 7: Informed prevalence (IP) estimates and corresponding 95% uncertainty intervals (UI) for porcine cysticercosis in Menoua division, Cameroon, 2000

Method	IP (%)	95% UI
Lingual	19	1–39
Ag(B158/B60)	6	2–11

The following code implements the multi-test model introduced by Berkvens et al. [47], yielding an informed prevalence of 14.0% (0.7–36.4).

```
PCC <-
  truePrevMulti(
    x = c(34, 9, 44, 620),
    n = 707,
    prior = {
      theta[1] ~ dunif(0.00, 0.50)
      theta[2] ~ dunif(0.161, 0.21)
      theta[3] ~ dunif(0.90, 1.00)
      theta[4] ~ dbeta(1, 1)
      theta[5] ~ dbeta(1, 1)
      theta[6] ~ dbeta(1, 1)
      theta[7] ~ dbeta(1, 1)
    }
  )
```

```
## show results
PCC

##       mean median  mode    sd  2.5% 97.5%
##  TP 0.140  0.113 0.059 0.103 0.007 0.364
## SE1 0.185  0.185 0.173 0.014 0.162 0.209
## SP1 0.959  0.956 0.949 0.019 0.927 0.997
## SE2 0.400  0.348 0.261 0.208 0.116 0.868
## SP2 0.925  0.922 0.905 0.030 0.876 0.985
##
## Multivariate BGR statistic = 1.007
## BGR values substantially above 1 indicate lack of convergence
## Bayes-P statistic = 0.5
## Bayes-P values substantially different from 0.5 indicate lack of convergence
```

```r
## density plots
par(mfcol = c(2, 4))
densplot(PCC, col = "red")
```

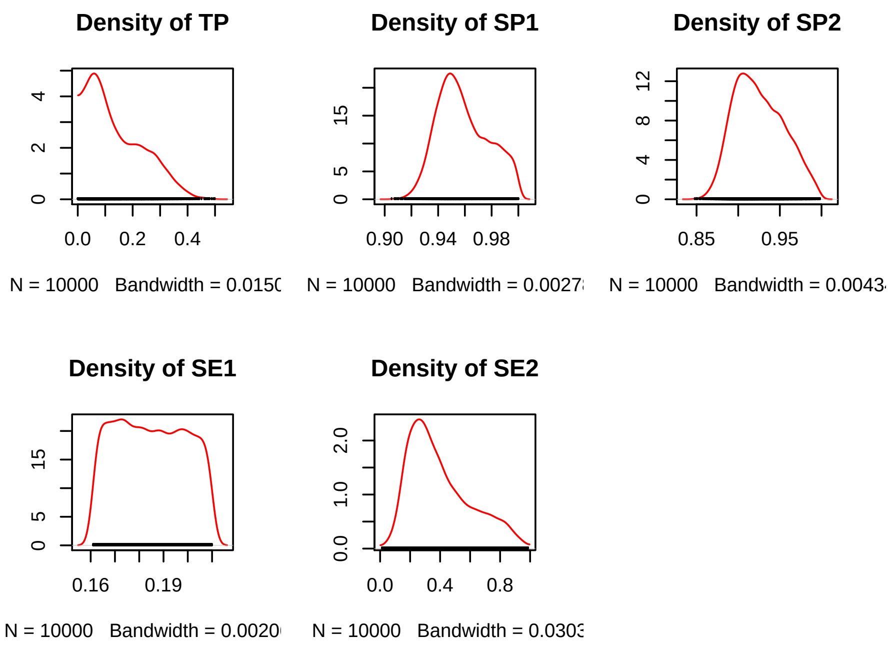


```r
## trace plots
par(mfcol = c(2, 4))
traceplot(PCC)
```

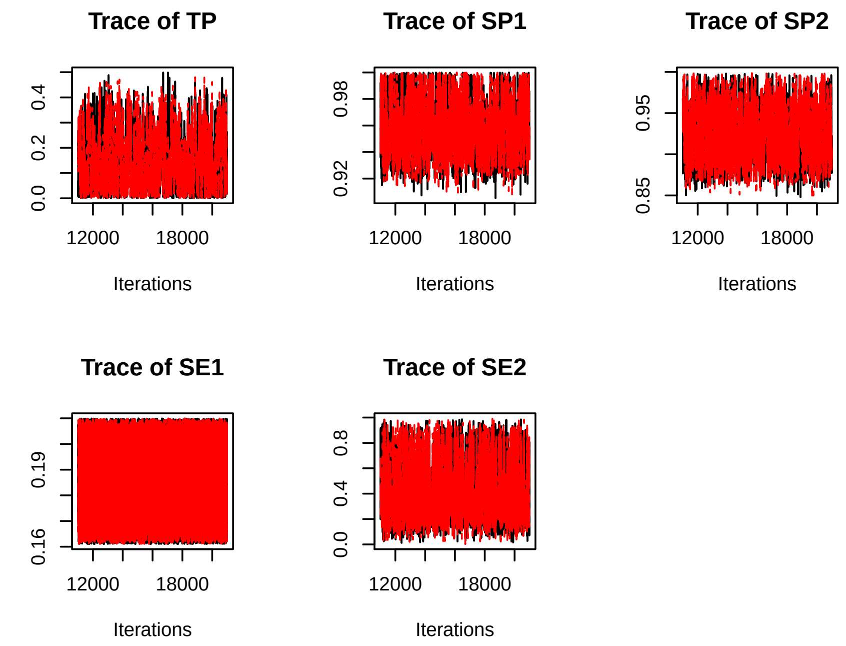

3.2.2 Krecek et al. 2008, 2011: Eastern Cape province, South Africa, 2003

In 2003, Krecek et al. [3, 4] examined 261 pigs in the Alfred Nzo and Oliver R. Tambo Districts of the Eastern Cape Province of South Africa. They used four tests: lingual examination, Ag-ELISA (HP10), Ag-ELISA (B158/B60) and Antibody EITB. In this analysis, only the results of the first three tests are considered. Table 8 shows the obtained joint test results.

Table 8: Results of the study on porcine cysticercosis in Eastern Cape province, South Africa, 2003 ($n = 261$) [3, 4]

Lingual examination	Ag-ELISA (HP10)	Ag-ELISA (B158/B60)	x
+	+	+	17
+	+	−	6
+	−	+	2
+	−	−	6
−	+	+	82
−	+	−	38
−	−	+	5
−	−	−	105

Table 9 shows the results of the informed prevalence assessments

Table 9: Informed prevalence (IP) estimates and corresponding 95% uncertainty intervals (UI) for porcine cysticercosis in Eastern Cape province, South Africa, 2003

Method	IP (%)	95% UI
Lingual	50	10–87
Ag(HP10)	54	5–97
Ag(B158/B60)	40	30–52

The following code implements the multi-test model introduced by Berkvens et al. [47], yielding an informed prevalence of 56.8% (47.1–72.5).

```
PCC <-
  truePrevMulti(
    x = c(17, 6, 2, 6, 82, 38, 5, 105),
    n = 261,
    prior = {
      theta[1] ~ dunif(0.00, 1.00)
      theta[2] ~ dunif(0.161, 0.210)
      theta[3] ~ dunif(0.900, 1.000)
      theta[4] ~ dunif(0.900, 1)  ## cf Krecek et al. 2008
      theta[5] ~ dbeta(1, 1)
      theta[6] ~ dunif(0.900, 1)  ## cf Krecek et al. 2008
      theta[7] ~ dbeta(1, 1)
      theta[8] ~ dunif(0.400, 1)  ## cf Krecek et al. 2008
      theta[9] ~ dbeta(1, 1)
      theta[10] ~ dbeta(1, 1)
      theta[11] ~ dbeta(1, 1)
```

```
      theta[12] ~ dunif(0.400, 1) ## cf Krecek et al. 2008
      theta[13] ~ dbeta(1, 1)
      theta[14] ~ dbeta(1, 1)
      theta[15] ~ dbeta(1, 1)
    }
)
```

```
## show results
PCC

##       mean median  mode    sd  2.5% 97.5%
##  TP 0.568  0.558 0.547 0.064 0.471 0.725
## SE1 0.180  0.178 0.165 0.013 0.162 0.207
## SP1 0.935  0.933 0.931 0.021 0.902 0.979
## SE2 0.917  0.936 0.969 0.066 0.741 0.989
## SP2 0.930  0.929 0.911 0.031 0.874 0.987
## SE3 0.663  0.668 0.674 0.060 0.528 0.765
## SP3 0.916  0.919 0.926 0.035 0.841 0.973
##
## Multivariate BGR statistic = 1.001
## BGR values substantially above 1 indicate lack of convergence
## Bayes-P statistic = 0.47
## Bayes-P values substantially different from 0.5 indicate lack of convergence
```

```
## density plots
par(mfcol = c(2, 4))
densplot(PCC, col = "red")
```

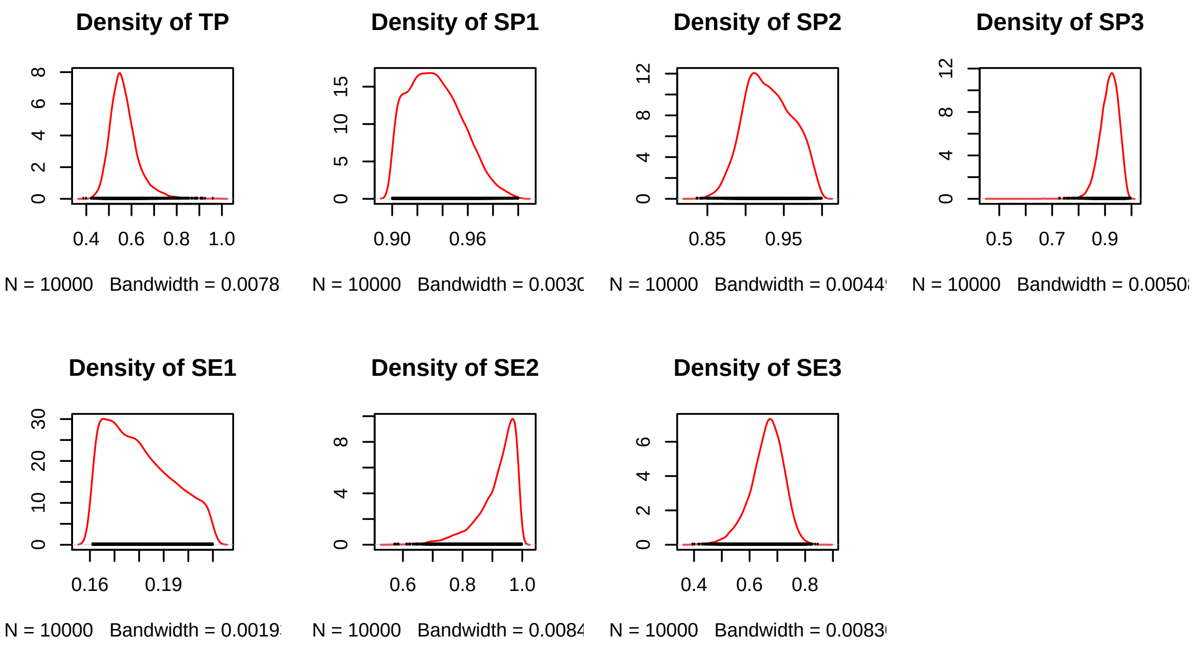

```r
## trace plots
par(mfcol = c(2, 4))
traceplot(PCC)
```

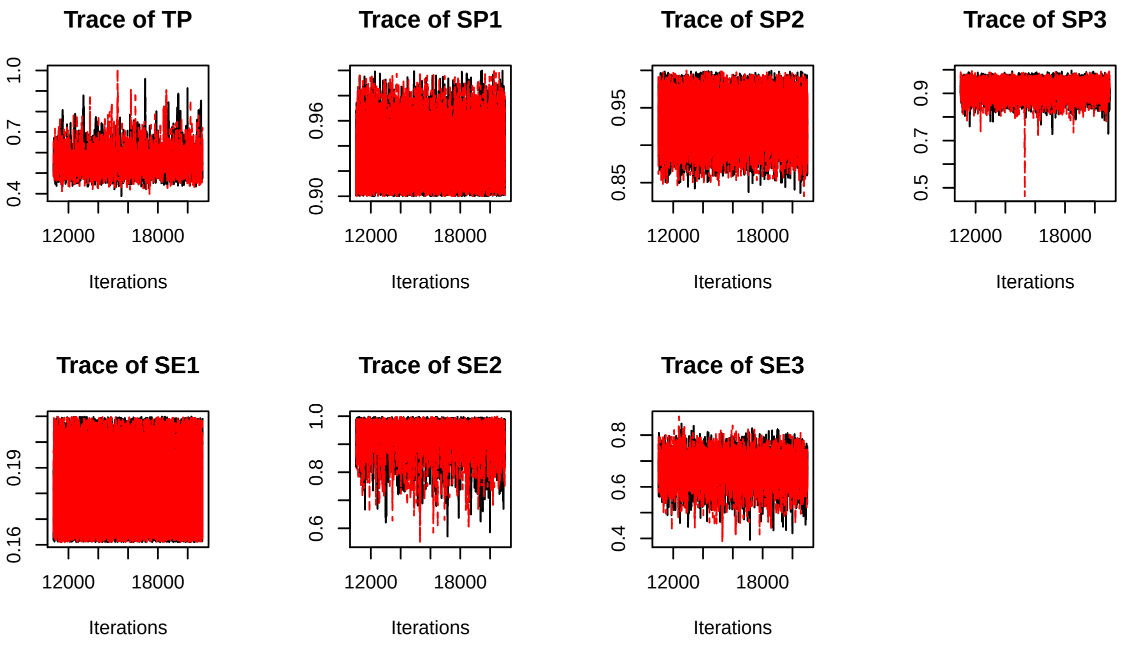

3.2.3 Dorny et al. 2004: Lusaka, Zambia, 2000

In 2000, Dorny et al. [2] examined 868 pigs in Lusaka, Zambia. They used four tests: lingual examination, post-mortem, Ag-ELISA (B158/B60) and Ab-ELISA. In this analysis, only the results of the first three tests are considered. Table 10 shows the obtained joint test results.

Table 10: Results of the study on porcine cysticercosis in Lusaka, Zambia, 2000 ($n = 868$) [2]

Lingual examination	Post-mortem	Ag-ELISA (B158/B60)	x
+	+	+	109
+	+	−	3
+	−	+	2
+	−	−	1
−	+	+	9
−	+	−	0
−	−	+	376
−	−	−	368

Table 11 shows the results of the informed prevalence assessments

Table 11: Informed prevalence (IP) estimates and corresponding 95% uncertainty intervals (UI) for porcine cysticercosis in Lusaka, Zambia, 2000

Method	IP (%)	95% UI
Lingual	59	28–86
Post-mortem	37	15–60
Ag(B158/B60)	56	46–67

The following code implements the multi-test model introduced by Berkvens et al. [47], yielding an informed prevalence of 73.3% (55.7–94.2).

```
PCC <-
  truePrevMulti(
    x = c(109, 3, 2, 1, 9, 0, 376, 368),
    n = 868,
    prior = {
      theta[1] ~ dunif(0.00, 1.00)
      theta[2] ~ dunif(0.161, 0.210)
      theta[3] ~ dunif(0.900, 1.000)
      theta[4] ~ dunif(0.221, 1)  ## cannot be worse than SE2
      theta[5] ~ dbeta(1, 1)
      theta[6] ~ dunif(0.900, 1)  ## cannot be worse than SP2
      theta[7] ~ dbeta(1, 1)
      theta[8] ~ dunif(0.645, 1)  ## cannot be worse than SE3
      theta[9] ~ dbeta(1, 1)
      theta[10] ~ dbeta(1, 1)
      theta[11] ~ dbeta(1, 1)
      theta[12] ~ dunif(0.912, 1) ## cannot be worse than SP3
      theta[13] ~ dbeta(1, 1)
      theta[14] ~ dbeta(1, 1)
      theta[15] ~ dbeta(1, 1)
    }
)
```

```
## show results
PCC

##      mean median  mode    sd  2.5% 97.5%
##  TP 0.733  0.729 0.733 0.100 0.557 0.942
## SE1 0.181  0.178 0.164 0.014 0.162 0.208
## SP1 0.975  0.981 0.994 0.021 0.919 0.999
## SE2 0.185  0.183 0.173 0.016 0.160 0.219
## SP2 0.958  0.963 0.975 0.027 0.896 0.996
## SE3 0.761  0.754 0.729 0.091 0.607 0.950
## SP3 0.920  0.921 0.913 0.034 0.852 0.982
##
## Multivariate BGR statistic = 1.003
## BGR values substantially above 1 indicate lack of convergence
## Bayes-P statistic = 0.63
## Bayes-P values substantially different from 0.5 indicate lack of convergence
```

```r
## density plots
par(mfcol = c(2, 4))
densplot(PCC, col = "red")
```

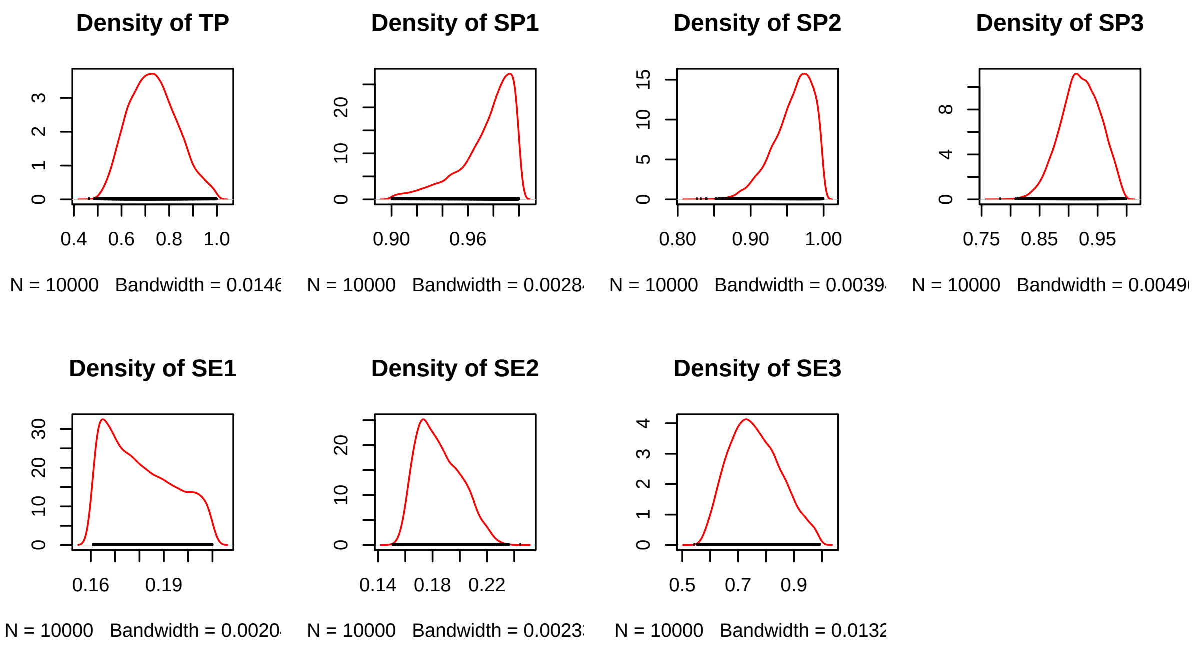


```r
## trace plots
par(mfcol = c(2, 4))
traceplot(PCC)
```

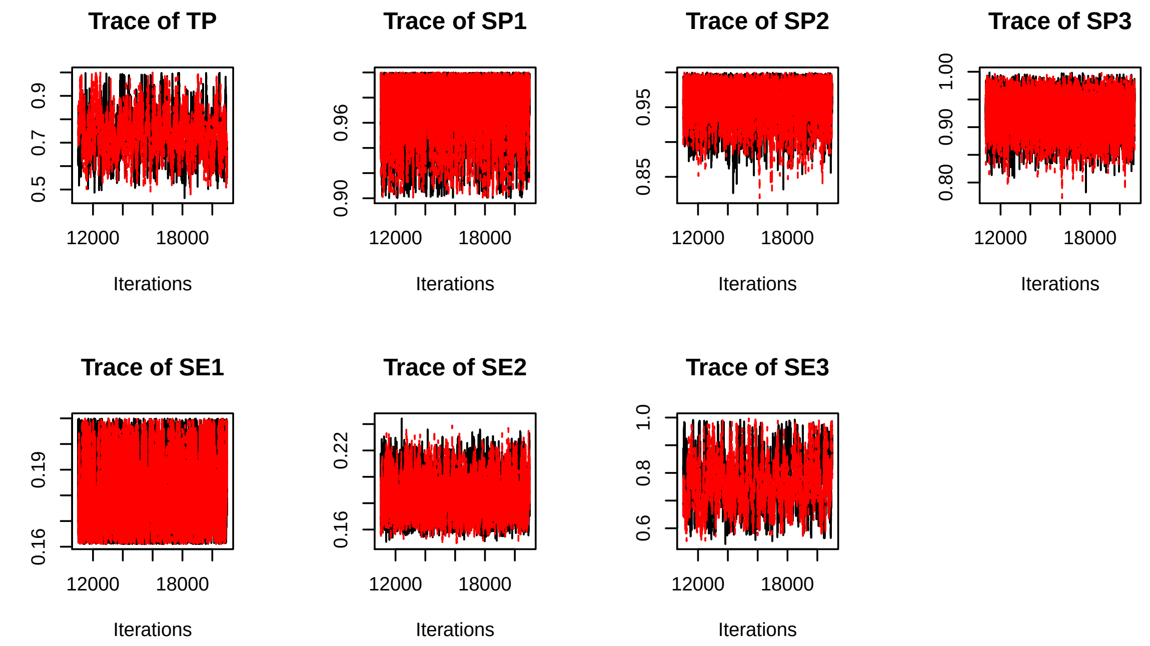

R Session Info

```
sessionInfo()

## R version 3.1.2 (2014-10-31)
## Platform: x86_64-w64-mingw32/x64 (64-bit)
##
## locale:
## [1] LC_COLLATE=Dutch_Belgium.1252  LC_CTYPE=Dutch_Belgium.1252
## [3] LC_MONETARY=Dutch_Belgium.1252 LC_NUMERIC=C
## [5] LC_TIME=Dutch_Belgium.1252
##
## attached base packages:
## [1] stats     graphics  grDevices utils     datasets  methods   base
##
## other attached packages:
## [1] prevalence_0.3.0    rjags_3-13          coda_0.16-1
## [4] lattice_0.20-29     xtable_1.7-1        XLConnect_0.2-9
## [7] XLConnectJars_0.2-9 knitr_1.6
##
## loaded via a namespace (and not attached):
## [1] evaluate_0.5.5 formatR_0.10   grid_3.1.2     highr_0.3
## [5] rJava_0.9-6    stringr_0.6.2  tools_3.1.2
```

References

[1] N. Praet, R. Rodriguez-Hidalgo, N. Speybroeck, S. Ahounou, W. Benitez-Ortiz, D. Berkvens, A. Van Hul, M. Barrionuevo-Samaniego, C. Saegerman, P. Dorny, Infection with versus exposure to *Taenia solium*: what do serological test results tell us?, The American Journal of Tropical Medicine and Hygiene 83 (2) (2010) 413–415.

[2] P. Dorny, I. Phiri, J. Vercruysse, S. Gabriël, A. Willingham III, J. Brandt, B. Victor, N. Speybroeck, D. Berkvens, A Bayesian approach for estimating values for prevalence and diagnostic test characteristics of porcine cysticercosis, International Journal for Parasitology 34 (5) (2004) 569–576.

[3] R. Krecek, L. Michael, P. Schantz, L. Ntanjana, M. Smith, P. Dorny, L. Harrison, F. Grimm, N. Praet, A. L. Willingham III, Prevalence of *Taenia solium* cysticercosis in swine from a community-based study in 21 villages of the Eastern Cape Province, South Africa, Veterinary Parasitology 154 (1) (2008) 38–47.

[4] R. Krecek, L. Michael, P. Schantz, L. Ntanjana, M. Smith, P. Dorny, L. Harrison, F. Grimm, N. Praet, A. Willingham III, Corrigendum to "Prevalence of *Taenia solium* cysticercosis in swine from a community-based study in 21 villages of the Eastern Cape Province, South Africa" [Vet. Parasitol. 154 (2008) 38–47], Veterinary Parasitology 183 (1) (2011) 198–200.

[5] N. Praet, J. J. Verweij, K. E. Mwape, I. K. Phiri, J. B. Muma, G. Zulu, L. Lieshout, R. Rodriguez-Hidalgo, W. Benitez-Ortiz, P. Dorny, Bayesian modelling to estimate the test characteristics of coprology, coproantigen ELISA and a novel real-time PCR for the diagnosis of taeniasis, Tropical Medicine & International Health 18 (5) (2013) 608–614.

[6] B. Devleesschauwer, P. Torgerson, J. Charlier, B. Levecke, N. Praet, S. Roelandt, S. Smit, P. Dorny, D. Berkvens, N. Speybroeck, prevalence: Tools for prevalence assessment studies., r package version 0.3.0 (2014).
URL http://cran.r-project.org/package=prevalence

[7] E. Newell, F. Vyungimana, S. Geerts, I. Van Kerckhoven, V. Tsang, D. Engels, Prevalence of cysticercosis in epileptics and members of their families in Burundi, Transactions of the Royal Society of Tropical Medicine and Hygiene 91 (4) (1997) 389–391.

[8] L. Vondou, A. Zoli, S. Pouedet, E. Assana, A. Kamga Tokam, P. Dorny, J. Brandt, S. Geerts, et al., La taeniose/cysticercose à *Taenia solium* dans la Menoua (Ouest-Cameroun), Parasite 9 (3) (2002) 271–274.

[9] K. Kanobana, N. Praet, C. Kabwe, P. Dorny, P. Lukanu, J. Madinga, P. Mitashi, M. Verwijs, P. Lutumba, K. Polman, High prevalence of *Taenia solium* cysticerosis in a village community of Bas-Congo, Democratic Republic of Congo, International Journal for Parasitology 41 (10) (2011) 1015–1018.

[10] H. Carstensen, H. L. Hansen, H. O. Kristiansen, G. Gomme, The epidemiology of cryptosporidiosis and other intestinal parasitoses in children in southern Guinea-Bissau, Transactions of the Royal Society of Tropical Medicine and Hygiene 81 (5) (1987) 860–864.

[11] S. Pampiglione, M. Ricciardi, S. Visconti, A. Branca, E. Olivieri, A. Zamberletti, Human intestinal parasites in Subsaharan Africa. I. Eastern Boe and Canhabaque Island (Guinea-Bissau), Parassitologia 29 (1) (1987) 1–13.

[12] M. Gweba, O. O. Faleke, A. U. Junaidu, J. P. Fabiyi, A. O. Fajinmi, Some risk factors for *Taenia solium* cysticercosis in semi-intensively raised pigs in Zuru, Nigeria, Veterinaria Italiana 46 (1) (2010) 57–67.

[13] A. Secka, F. Grimm, T. Marcotty, D. Geysen, A. M. Niang, V. Ngale, L. Boutche, E. Van Marck, S. Geerts, Old focus of cysticercosis in a Senegalese village revisited after half a century, Acta Tropica 119 (2) (2011) 199–202.

[14] M. Pammenter, E. Rossouw, C. Dingle, Serological detection of cysticercosis in two rural areas of South Africa, Transactions of the Royal Society of Tropical Medicine and Hygiene 81 (2) (1987) 242–244.

[15] K. S. Eom, J.-Y. Chai, T.-S. Yong, D.-Y. Min, H.-J. Rim, C. Kihamia, H.-K. Jeon, Morphologic and genetic identification of *Taenia* tapeworms in Tanzania and DNA genotyping of *Taenia solium*, The Korean Journal of Parasitology 49 (4) (2011) 399–403.

[16] G. Mwanjali, C. Kihamia, D. V. C. Kakoko, F. Lekule, H. Ngowi, M. V. Johansen, S. M. Thamsborg, A. L. Willingham III, Prevalence and risk factors associated with human *Taenia solium* infections in Mbozi District, Mbeya Region, Tanzania, PLoS Neglected Tropical Diseases 7 (3) (2013) e2102.

[17] N. Kabatereine, J. Kemijumbi, F. Kazibwe, A. Onapa, Human intestinal parasites in primary school children in Kampala, Uganda, East African Medical Journal 74 (5) (1997) 311–314.

[18] K. E. Mwape, I. K. Phiri, N. Praet, J. B. Muma, G. Zulu, P. Van den Bossche, R. De Deken, N. Speybroeck, P. Dorny, S. Gabriël, *Taenia solium* infections in a rural area of Eastern Zambia—a community based study, PLoS Neglected Tropical Diseases 6 (3) (2012) e1594.

[19] R. Ganaba, N. Praet, H. Carabin, A. Millogo, Z. Tarnagda, P. Dorny, S. Hounton, A. Sow, P. Nitiéma, L. D. Cowan, Factors associated with the prevalence of circulating antigens to porcine cysticercosis in three villages of Burkina Faso, PLoS Neglected Tropical Diseases 5 (1) (2011) e927.

[20] E. Assana, P. Zoli, H. Sadou, L. Vondou, M. Pouedet, P. Dorny, J. Brandt, S. Geerts, Prévalence de la cysticercose porcine dans le Mayo-Danay (nord Cameroun) et le Mayo-Kebbi (sud-ouest du Tchad), Revue d'élevage et de médecine vétérinaire des pays tropicaux 54 (2) (2001) 123–127.

[21] E. Assana, F. Amadou, E. Thys, M. Lightowlers, A. Zoli, P. Dorny, S. Geerts, Pig-farming systems and porcine cysticercosis in the north of Cameroon, Journal of Helminthology 84 (4) (2010) 441–446.

[22] N. A. N. Ngwing, J. W. Poné, M. Mbida, A. Z. Pagnah, H. Njakoi, C. Bilong, A preliminary analysis of some epidemiological factors involved in porcine cysticercosis in Bafut and Santa subdivisions, North West Region of Cameroon, Asian Pacific Journal of Tropical Medicine 5 (10) (2012) 814–817.

[23] M. Pouedet, A. Zoli, L. Vondou, E. Assana, N. Speybroeck, D. Berkvens, P. Dorny, J. Brandt, S. Geerts, Epidemiological survey of swine cysticercosis in two rural communities of West-Cameroon, Veterinary Parasitology 106 (1) (2002) 45–54.

[24] O. Shey-Njila, P. Zoli, J. Awah-Ndukum, E. Assana, P. Byambas, P. Dorny, J. Brandt, S. Geerts, Porcine cysticercosis in village pigs of North-West Cameroon, Journal of Helminthology 77 (4) (2003) 351–354.

[25] N. Praet, K. Kanobana, C. Kabwe, V. Maketa, P. Lukanu, P. Lutumba, K. Polman, P. Matondo, N. Speybroeck, P. Dorny, *Taenia solium* cysticercosis in the Democratic Republic of Congo: how does pork trade affect the transmission of the parasite?, PLoS Neglected Tropical Diseases 4 (9) (2010) e817.

[26] A. Permin, L. Yelifari, P. Bloch, N. Steenhard, N. Hansen, P. Nansen, Parasites in cross-bred pigs in the Upper East Region of Ghana, Veterinary Parasitology 87 (1) (1999) 63–71.

[27] E. E. Eshitera, S. M. Githigia, P. Kitala, L. F. Thomas, E. M. Fèvre, L. J. Harrison, E. W. Mwihia, R. O. Otieno, F. Ojiambo, N. Maingi, Prevalence of porcine cysticercosis and associated risk factors in Homa Bay District, Kenya, BMC Veterinary Research 8 (1) (2012) 234.

[28] S. Githigia, A. Murekefu, R. Otieno, Prevalence of porcine cysticercosis and risk factors for *Taenia solium* taeniosis in Funyula Division of Busia District, Kenya, Kenya Veterinarian 29 (1) (2007) 37–39.

[29] J. Kagira, N. Maingi, P. Kanyari, S. Githigia, J. Ng'ang'a, J. Gachohi, Seroprevalence of *Cysticercus cellulosae* and associated risk factors in free-range pigs in Kenya, Journal of Helminthology 84 (4) (2010) 398–403.

[30] F. K. Mutua, T. F. Randolph, S. M. Arimi, P. M. Kitala, S. M. Githigia, A. L. Willingham, F. M. Njeruh, Palpable lingual cysts, a possible indicator of porcine cysticercosis, in Teso District, Western Kenya, Journal of Swine Health and Production 15 (4) (2007) 206.

[31] A. Pondja, L. Neves, J. Mlangwa, S. Afonso, J. Fafetine, A. L. Willingham III, S. M. Thamsborg, M. V. Johansen, Prevalence and risk factors of porcine cysticercosis in Angonia District, Mozambique, PLoS Neglected Tropical Diseases 4 (2) (2010) e594.

[32] A. A. Biu, J. Ijudai, Prevalence and morphometric studies on porcine cysticercosis in Adamawa State, Nigeria, Sokoto Journal of Veterinary Sciences 10 (1) (2012) 28–31.

[33] N. Karshima, A. Bobbo, A. Udokainyang, A. Salihu, *Taenia solium* cysticercosis in pigs slaughtered in Ibi local government area of Taraba state, Nigeria, Journal of Animal Science Advances 3 (3) (2013) 109–113.

[34] D. Onah, S. Chiejina, *Taenia solium* cysticercosis and human taeniasis in the Nsukka area of Enugu State, Nigeria, Annals of Tropical Medicine & Parasitology 89 (4) (1995) 399–407.

[35] M. Boa, H. Bøgh, A. Kassuku, P. Nansen, The prevalence of *Taenia solium* metacestodes in pigs in northern Tanzania, Journal of Helminthology 69 (2) (1995) 113–117.

[36] M. Boa, E. Mahundi, A. Kassuku, A. Willingham III, N. C. Kyvsgaard, Epidemiological survey of swine cysticercosis using ante-mortem and post-mortem examination tests in the southern highlands of Tanzania, Veterinary Parasitology 139 (1) (2006) 249–255.

[37] U. C. Braae, P. Magnussen, F. Lekule, W. Harrison, M. V. Johansen, Temporal fluctuations in the sero-prevalence of *Taenia solium* cysticercosis in pigs in Mbeya Region, Tanzania, Parasites & Vectors 7 (1) (2014) 574.

[38] E. V. Komba, E. C. Kimbi, H. A. Ngowi, S. I. Kimera, J. E. Mlangwa, F. P. Lekule, C. S. Sikasunge, A. L. Willingham III, M. V. Johansen, S. M. Thamsborg, Prevalence of porcine cysticercosis and associated risk factors in smallholder pig production systems in Mbeya region, southern highlands of Tanzania, Veterinary Parasitology 198 (3) (2013) 284–291.

[39] E. M. Mkupasi, H. A. Ngowi, H. E. Nonga, Prevalence of extra-intestinal porcine helminth infections and assessment of sanitary conditions of pig slaughter slabs in Dar es Salaam city, Tanzania, Tropical Animal Health and Production 43 (2) (2011) 417–423.

[40] H. Ngowi, A. Kassuku, G. Maeda, M. Boa, H. Carabin, A. Willingham III, Risk factors for the prevalence of porcine cysticercosis in Mbulu District, Tanzania, Veterinary Parasitology 120 (4) (2004) 275–283.

[41] C. Yohana, C. Mwita, G. Nkwengulila, The prevalence of porcine cysticercosis and risk factors for taeniasis in Iringa rural district, International Journal of Animal and Veterinary Advances 5 (6) (2013) 251–255.

[42] C. Waiswa, E. Fèvre, Z. Nsadha, C. Sikasunge, A. Willingham, Porcine cysticercosis in southeast Uganda: seroprevalence in Kamuli and Kaliro districts, Journal of Parasitology Research 2009 (2009) 375493.

[43] I. Phiri, P. Dorny, S. Gabriël, A. Willingham III, N. Speybroeck, J. Vercruysse, The prevalence of porcine cysticercosis in Eastern and Southern provinces of Zambia, Veterinary Parasitology 108 (1) (2002) 31–39.

[44] I. Phiri, P. Dorny, S. Gabriël, A. L. Willingham, C. Sikasunge, S. Siziya, J. Vercruysse, Assessment of routine inspection methods for porcine cysticercosis in Zambian village pigs, Journal of Helminthology 80 (1) (2006) 69–72.

[45] C. S. Sikasunge, I. K. Phiri, A. M. Phiri, S. Siziya, P. Dorny, A. L. Willingham III, Prevalence of *Taenia solium* porcine cysticercosis in the Eastern, Southern and Western provinces of Zambia, The Veterinary Journal 176 (2) (2008) 240–244.

[46] N. Speybroeck, B. Devleesschauwer, L. Joseph, D. Berkvens, Misclassification errors in prevalence estimation: Bayesian handling with care, International Journal of Public Health 58 (2013) 791–795.

[47] D. Berkvens, N. Speybroeck, N. Praet, A. Adel, E. Lesaffre, Estimating disease prevalence in a Bayesian framework using probabilistic constraints, Epidemiology 17 (2) (2006) 145–153.
